# Cirrhosis outcomes on rurality and weekend admissions revisited: A contemporary analysis of the national inpatient sample

**DOI:** 10.1371/journal.pone.0353178

**Published:** 2026-07-02

**Authors:** Ahamed Khalyfa, Betty H. Tu, Qianyi Shi, Tomohiro Tanaka

**Affiliations:** 1 Division of Gastroenterology and Hepatology, University of Iowa, Iowa City, Iowa, United States of America; 2 University of Iowa Carver College of Medicine, Iowa City, Iowa, United States of America; 3 University of Iowa College of Public Health, Iowa City, Iowa, United States of America; University Hospital of Bologna Sant’Orsola-Malpighi Polyclinic Department of Digestive System: Azienda Ospedaliero-Universitaria di Bologna Policlinico Sant’Orsola-Malpighi Dipartimento dell’apparato digerente, ITALY

## Abstract

**Background and Aims:**

The impact of hospital rurality and weekend admission on outcomes in decompensated cirrhosis remains unclear. Studies suggest mixed effects of weekend admission on mortality and increased mortality in rural hospital admissions for decompensated cirrhosis. This study evaluated the influence of hospital rurality, weekend admission, and their interaction on outcomes in decompensated cirrhosis from 2016 to 2020.

**Methods:**

A cross-sectional analysis of the National Inpatient Sample (NIS) assessed in-hospital mortality (primary outcome), likelihood of specific procedures (esophagogastroduodenoscopy, paracentesis, TIPS, hemodialysis), and time to first procedure. Regression models adjusted for demographics, liver disease etiology, clinical severity (APR-DRG mortality risk), and other factors.

**Results:**

Among 11,845,223 hospitalizations, rural hospitalizations were linked to lower in-hospital mortality (OR: 0.84; 95% CI: 0.80–0.86) and higher transfer rates for severe cases (7.2% vs. 2.8%, p < 0.001). Weekend admissions showed a statistically significant but only modest reduction in mortality odds (OR: 0.99; 95% CI: 0.975–0.998). No significant interaction existed between rurality and weekend admission regarding mortality. Rural hospitals showed lower odds of performing procedures (95% CIs < 1), though time to procedure was comparable, except for earlier hemodialysis (−1.35 days; 95% CI: −2.59 to −0.11). Weekend admissions did not significantly impact procedure rates, except for paracentesis (OR: 0.94; 95% CI: 0.88–0.99).

**Conclusions:**

Using a national cohort of hospitalized patients with decompensated cirrhosis, we showed that rural hospitals exhibited lower in-hospital mortality, fewer procedures, and higher transfer rates, and that weekend admissions showed only a minimal, clinically insignificant reduction in mortality, irrespective of hospital rurality.

## Introduction

Chronic liver disease (CLD) and cirrhosis have consistently been significant public health concerns, experiencing a significant increase in death rates during the COVID-19 pandemic and ranking among the leading causes of mortality in the United States [1,2]. Cirrhosis arises from diverse etiologies including chronic viral hepatitis (hepatitis B and C), metabolic factors (obesity, diabetes, hyperlipidemia), autoimmune disorders, and dietary factors (alcohol, high-fat diet) [3]. Decompensated cirrhosis occurs when complications related to liver dysfunction and/or portal hypertension, such as ascites, hepatic encephalopathy, or variceal hemorrhage, arise. These complications are associated with high mortality, underscoring the need for prompt evaluation and management [4].

Cirrhosis outcomes are influenced by both various factors within the healthcare system, including the timing of hospital admission and hospital location, which have been examined independently in prior studies. Regarding timing, weekend admissions have been associated with higher rebleeding rates with variceal bleeding [5] and lower rates of endoscopic intervention for upper gastrointestinal hemorrhage (UGIH) [6] than weekday admissions—though the impact of weekend admission on overall mortality in cirrhosis is inconsistent across studies [1]. Regarding hospital location, rural hospitals encounter greater systemic difficulties than urban hospitals, including declining inpatient volumes, financial constraints, and increased rates of closure [7]. Prior analyses have indicated higher in-hospital mortality among patients with end-stage liver disease admitted to rural hospitals, independent of disease severity [6].

While prior studies have examined the independent effects of weekend admissions and hospital rurality on cirrhosis outcomes, their combined interaction has not been investigated. This study aims to address the gap by exploring the combined impact of hospital rurality and weekend admission on outcomes in hospitalized patients with decompensated cirrhosis using a contemporary national cohort dataset from 2016–2020.

## Patients and methods

We conducted a cross-sectional study of patients hospitalized with cirrhosis from 2016 to 2020, using the national inpatient sample (NIS) database. The NIS annually contains all-payer data, including approximately 7 million hospital discharges from around 4,500 hospitals across most states (47 states in 2016, 48 in 2017–2018, and 49 in 2019–2020). The NIS is a self-weighted, stratified, systematic, random sample of discharges from all Healthcare Cost and Utilization Project (HCUP) hospitals. Hospitals are stratified by census division, location and teaching status, bed size category, and ownership. Each sampled discharge is assigned a weight that represents the number of universe discharges within its stratum for that year. The NIS samples approximately 20% of universe discharges in each stratum, resulting in discharge weights that typically approach a value of five. This study is reported following the Strengthening the Reporting of Observational Studies in Epidemiology (STROBE) reporting guidelines. Data were accessed in the NIS between 11/8/2023–5/28/2024.

Our primary objective was to determine the association between the hospital-level rurality, weekend admissions, and their interaction on the following hospital outcomes: mortality (primary outcome); chances to receive procedures such as upper endoscopy (EGD), transjugular intrahepatic portosystemic shunt (TIPS), and hemodialysis (HD); and days to receipt of these procedures. In-hospital mortality, defined as death occurring during the index hospitalization at the admitting facility, served as the primary outcome; because the NIS does not link records across facilities, deaths occurring after transfer to another hospital are attributed to the receiving institution’s record and cannot be traced back to the originating admission. Potential confounders included sex, age, race, etiology of liver disease, patient-level rurality, APR-DRG risk of mortality subclass (a measure of clinical severity), and socioeconomic factors including insurance status (Medicaid or other insurance), hospital region in the United States (Northeast, Midwest, South, West), and estimated median household income based on ZIP code.

Our study focused on a specific subpopulation within the NIS: adult patients diagnosed with decompensated cirrhosis who were hospitalized between 2016 and 2020. We included all individuals age ≥ 18 years with at least one cirrhosis-related ICD-10-CM code or at least one cirrhosis-related complication ICD-10-CM code (i.e., ascites, hepatic encephalopathy, variceal hemorrhage, and hepatorenal syndrome [HRS]). The list of ICD-10 codes used in the study is listed in S1 Table. Analyzing subgroups within datasets collected through complex survey design, like the NIS, requires special consideration to maintain the integrity of variance estimates. Simply excluding non-relevant individuals from the NIS could lead to inaccurate standard errors, particularly if this process eliminates entire hospitals from the sample. To maintain the statistical integrity of the complex survey design, subpopulation analyses were conducted using the HCUP-recommended ‘dummy observation’ approach. This method ensures that the full sampling structure, including all strata and primary sampling units (PSUs), is preserved during variance estimation. By retaining the original design rather than simple subset deletion, we avoid underestimating standard errors and ensure that the degrees of freedom correctly reflect the National Inpatient Sample’s complex architecture [8].

The unit of analysis was each unique hospital admission. For mortality, we analyzed the subset of the full NIS sample based on our population of interest (i.e., adult patients with decompensated cirrhosis who were hospitalized between 2016 and 2020). For receiving upper endoscopy, variceal ligation or other control of bleeding, or TIPS procedures, we analyzed the subset of the study population with variceal bleeding; for those receiving hemodialysis, we analyzed the subset of the study population with hepatorenal syndrome; and for those receiving paracentesis, we analyzed the subset of the study population with spontaneous bacterial peritonitis.

For categorical variables, proportions were reported, and χ2 tests were used to examine differences in distribution by rurality. For continuous variables, median, interquartile range (IQR), minimum, and maximum values were reported. Wilcoxon rank-sum tests for complex survey samples were used to examine differences in distribution by rurality [9].

Multivariable logistic regression models were employed to estimate the odds ratios (OR) for in-hospital mortality, while multivariable linear regression was used for length of stay (LOS). To evaluate whether the ‘weekend effect’ differed by hospital location, an interaction term between hospital rurality (rural vs. urban) and admission timing (weekend vs. weekday) was included in the models. The significance of the interaction was assessed using the Wald test. To ensure the robustness of our findings and address potential survival bias, we performed several sensitivity analyses. We first excluded all patient transfers to ensure the lower rural mortality was not simply a byproduct of triaging high-acuity patients to urban centers. We then conducted an acuity-stratified analysis, categorizing patients into ‘Low-to-Moderate’ (APR-DRG 1–2) and ‘Severe-to-Extreme’ (APR-DRG 3–4) mortality risk (both transfer-inclusive and exclusive). Finally, we confirmed the stability of our estimates by using a restrictive ICD-10 definition for cirrhosis and excluding the 2020 calendar year to account for COVID-19-related disruptions.

All the analyses incorporated sampling weights and were conducted using R statistical software version 4.1.2 and the Survey (Version 4.4−2; Lumley, 2024) package. All statistical tests were two-sided, with significance set at p-value < 0.05.

**Ethics Statement:** The Institutional Review Board of the University of Iowa reviewed this study and determined it to be exempt from further oversight because it involved analysis of de-identified data from the National Inpatient Sample (reference number 202510384).

**Patient consent statement:** As the NIS database lacks patient-specific and hospital-specific identifiers, this study did not require informed consent.

## Results

### Demographics

**Fig 1:**
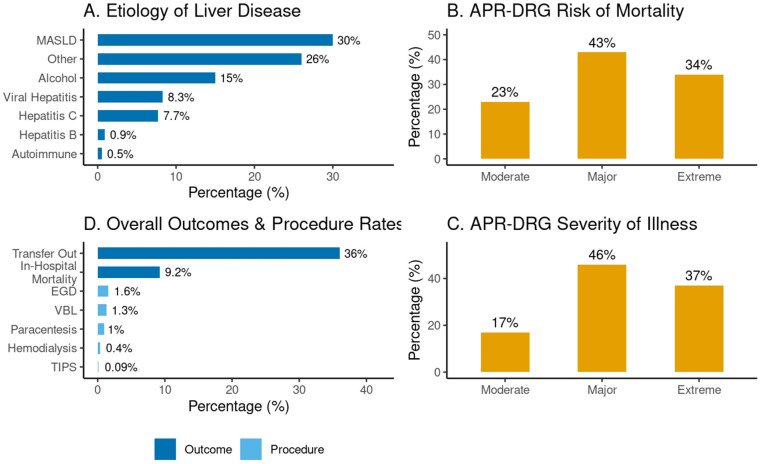
Cohort Overview of Hospital Admissions for Decompensated Cirrhosis, 2016-2020. (A) Distribution of liver disease etiology. (B) APR-DRG risk of mortality subclass. (C) APR-DRG severity of illness subclass. (D) Overall in-hospital outcomes and procedure utilization rates. Data derived from the National Inpatient Sample (NIS); N = 11,845,223.

Baseline characteristics for the study population are summarized in Table 1 and Fig 1 for key clinical trends. A total of 11,845,223 hospitalizations for decompensated cirrhosis were analyzed between 2016 and 2020. The median patient age was 68 years. Regarding race, 67% (7,905,424) of patients were White, 14% (1,658,060) were Black, 11% (1,264,375) were Hispanic, and 8.6% (1,017,365) identified as other races. Nearly half (48%) of the patients were female. The most common etiology of cirrhosis was metabolic dysfunction-associated steatotic liver disease (MASLD) (30%), followed by other etiologies (26%), alcohol-related liver disease (15%), viral hepatitis (excluding hepatitis B or C) (8.3%), hepatitis C (7.7%), hepatitis B (0.9%), and autoimmune hepatitis (0.5%). Most hospitalizations occurred in the South (41%), followed by the Midwest (22%), the West (21%), and the Northeast (16%). Regarding APR-DRG risk of mortality subclasses, 23% had a moderate likelihood of dying, 46% of patients had a major likelihood of dying, and 34% had an extreme likelihood.

**Table 1:** Characteristics of hospital admissions for decompensated cirrhosis between 2016 and 2020.

Characteristics	N/Mean	(%)/SD
**Total Admissions**	11,845,223	**–**
Rural admissions	872,125	7.4%
Urban admissions	10,973,098	93%
Weekend admissions	2,879,570	24%
**Demographics**		
Age (years)	66.70	15.44
Sex (female)	5,708,480	48%
Race		
White	7,905,424	67%
Black	1,658,060	14%
Hispanic	1,264,375	11%
Other	11,017,365	8.6%
Insurance (Medicaid)	1,789,939	15%
Region		
Northeast	1,905,340	16%
Midwest	2,549,111	22%
South	4,848,116	41%
West	2,542,656	21%
**Etiology of Liver Disease**		
Alcohol	1,725,509	15%
MASLD	3,573,659	30%
Hepatitis C	908,315	7.7%
Hepatitis B	104,740	0.9%
Autoimmune	55,590	0.5%
Viral hepatitis	982,350	8.3%
Other etiology	3,061,124	26%
**Cirrhosis Complications**		
Variceal hemorrhage	221,440	1.9%
Ascites	2,582,449	22%
Spontaneous bacterial peritonitis	174,670	1.5%
Hepatorenal syndrome	223,580	1.9%
**APR-DRG Risk of Mortality**		
Moderate	2,698,334	23%
Major	5,066,410	43%
Extreme	4,080,480	34%
**APR-DRG Severity of Illness**		
Moderate	2,017,625	17%
Major	5,408,094	46%
Extreme	4,419,505	37%
**In-hospital mortality**	1,090,220	9.2%
**Length of stay** (days)	8.05	10.29
**Transfer out of hospital**	4,297,055	36%
To acute care centers	366,530	3.1%
To other healthcare centers	3,930,525	33%
**Procedures Received**		
Hemodialysis for HRS	48,830	0.4%
Paracentesis for SBP	121,240	1.0%
TIPS for VH	10,550	<0.1%
All EGD for VH	188,030	1.6%
EGD with VBL for VH	148,590	1.3%
**Days to Procedures Received**		
Hemodialysis for HRS	5.26	7.19
Paracentesis for SBP	2.22	4.63
TIPS for VH	2.67	3.63
All EGD for VH	1.24	2.51
EGD with VBL for VH	1.24	2.56

Data on hospital admissions was obtained from the National Inpatient Sample (NIS) database.

Abbreviations: APR-DRG = all patient refined diagnosis related group; MASLD = metabolic dysfunction-associated steatotic liver disease; SBP = spontaneous bacterial peritonitis; SD = standard deviation, TIPS = transjugular intrahepatic portosystemic shunt; VH = variceal hemorrhage.

Stratification of demographics by rural versus urban location and weekend versus weekday admission revealed no significant differences in age, gender distribution, or cirrhosis etiology (S2 Table). A higher proportion of patients had Medicaid during weekend admissions in urban areas (16%) compared to rural areas (13%). Regionally, a higher proportion of rural hospitalizations were located in the South (51%) compared to urban hospitalizations (40%).

### Crude outcomes by rurality and weekend admission

#### Mortality.

**Fig 2:**
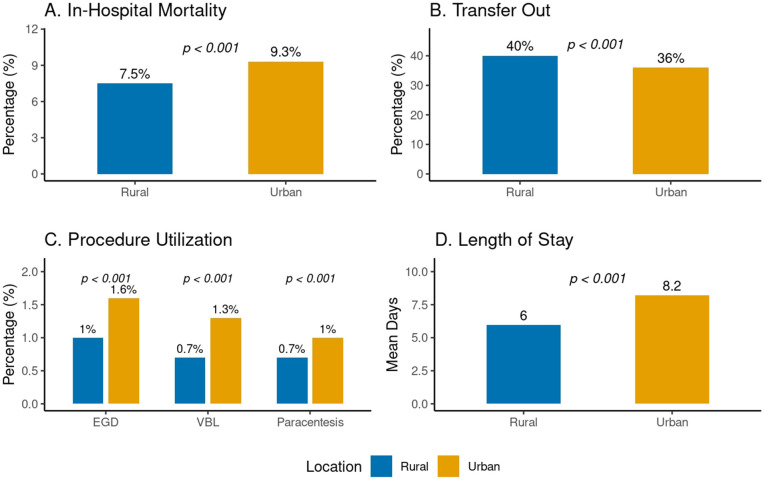
Key clinical Outcomes and Procedure Utilization by Hospital Rurality. (A) In-hospital mortality. (B) Transfer rates. (C) EGD, Variceal band ligation (VBL), and paracentesis utilization. (D) Mean length of stay.

A total of 1,090,220 patients died during hospitalization (Table 1). Urban hospitals accounted for 1,025,185 deaths (9.3% of urban hospitalizations), while 65,035 deaths (7.5% of rural hospitalizations) occurred in rural hospitals (p < 0.001) (Table 2, Fig 2). Within urban hospitals, mortality after weekday admissions was higher (767,940 deaths or 9.3%) compared to mortality after weekend admissions (257,240 deaths or 9.6%) (p < 0.001). In rural hospitals, weekday admissions (48,820 deaths or 7.4%) showed a trend toward higher mortality than weekend admissions (16,215 deaths or 7.7%) (p = 0.062).

**Table 2:** Select characteristics by rurality and weekend admission of hospital admissions for decompensated cirrhosis between 2016 and 2020.

Characteristics	Total Urban admissions		Total Rural admissions		p-value	Urban admissions					Rural admissions				
**Urban/Weekday** **admissions**		**Urban/Weekend** **admissions**		**p-value**	**Rural/Weekday** **admissions**		**Rural/Weekend** **admissions**		**p-value**
N/Mean	(%)/SD	N/Mean	(%)/SD		N/Mean	(%)/SD	N/Mean	(%)/SD		N/Mean	(%)/SD	N/Mean	(%)/SD
**Number of admissions**	10,973,098	93%	872,125	7.4%		8,305,004	76%	2,668,075	24%		660,630	76%	211,495	24%	
**In-hospital mortality**	1,025,185	9.3%	65,035	7.5%	<0.001	767,940	9.3%	257,240	9.6%	<0.001	48,820	7.4%	16,215	7.7%	0.062
**Length of stay** (days)	8.21	10.52	5.95	6.46	<0.001	8.27	10.55	8.05	10.42	<0.001	6.01	6.61	5.71	5.94	<0.001
**Transfer out of hospital**	3,945,609	36%	351,445	40%	<0.001	2,966,514	36%	979,090	37%	<0.001	264,545	40%	86,900	41%	<0.001
**Procedures Received**															
Hemodialysis for HRS	47,745	0.4%	1,085	0.1%	<0.001	36,835	0.4%	10,910	0.4%	0.001	850	0.1%	235	0.1%	0.4
Paracentesis for SBP	114,970	1.0%	6,270	0.7%	<0.001	87,045	1.0%	27,925	1.0%	>0.9	4,860	0.7%	1,410	0.7%	0.14
TIPS for VH	10,455	<0.1%	95	<0.1%	<0.001	7,790	<0.1%	2,665	<0.1%	0.2	75	<0.1%	20	<0.1%	0.7
All EGD for VH	179,660	1.6%	8,370	1.0%	<0.001	133,155	1.6%	46,505	1.7%	<0.001	6,415	1.0%	1,955	0.9%	0.4
EGD with VBL for VH	142,550	1.3%	6,040	0.7%	<0.001	105,405	1.3%	37,145	1.4%	<0.001	4,610	0.7%	1,430	0.7%	0.6
**Days to Procedures Received**															
Hemodialysis for HRS	5.29	7.24	3.78	4.32	0.023	5.35	7.41	5.10	6.63	0.5	3.99	4.52	2.97	3.34	0.3
Paracentesis for SBP	2.24	4.71	1.67	2.91	<0.001	2.23	4.96	2.29	3.79	<0.001	1.65	3.07	1.75	2.30	0.011
TIPS for VH	2.66	3.61	4.33	5.37	0.3	2.59	3.71	2.86	3.32	<0.001	4.15	5.60	5.50	4.95	0.2
All EGD for VH	1.25	2.53	1.09	1.79	0.4	1.24	2.60	1.26	2.33	<0.001	1.05	1.81	1.24	1.74	0.002
EGD with VBL for VH	1.25	2.58	1.05	1.89	0.043	1.25	2.63	1.26	2.43	<0.001	1.00	1.88	1.20	1.91	<0.001

Abbreviations: APR-DRG = all patient refined diagnosis related group; IQR = interquartile range; MASLD = metabolic dysfunction-associated steatotic liver disease; SBP = spontaneous bacterial peritonitis; SD = standard deviation.

#### Length of stay (LOS).

Overall, the median length of stay (LOS) was 5 days (SD: 10) **(**Table 1). Patients in rural hospitals had shorter stays compared to urban hospitals (mean 5.95 vs 8.21 days, median 4 vs. 5 days, p < 0.001) (Table 2, Fig 2). There were no significant differences in LOS between weekend and weekday admissions.

#### Transfers to tertiary or acute care centers.

Of all the 4,297,055 transfers, 366,530 were to acute care centers, and 3,930,525 were to other healthcare facilities **(**Table 1). Overall, 36% of urban admissions resulted in a transfer, compared to 40% in rural admissions (p < 0.001), indicating a higher likelihood of transfer when individuals were hospitalized in rural hospitals (Table 2, Fig 2). This trend was consistent across both weekday and weekend admissions. Weekend admissions statistically significantly affected transfer rates, though the effect size was not substantial in either urban (37% vs. 36%, p < 0.001) or rural (41% vs. 40%, p < 0.001) settings (Table 2).

#### Admission diagnosis and procedures received.

The most common decompensated event at admission was ascites (22%), followed by hepatorenal syndrome (1.9%), variceal hemorrhage (1.9%), and spontaneous bacterial peritonitis (SBP) (1.5%) (Table 1). Urban hospitals had higher rates of SBP, variceal hemorrhage, ascites, and hepatorenal syndrome compared to rural hospitals (all p < 0.001) (Table 2, Fig 2).

Among urban admissions, hospitalizations during weekdays were associated with higher proportions of ascites (23% vs. 21%, p < 0.001) and hepatorenal syndrome (1.9% vs. 1.8%, p < 0.001) compared to weekend admissions. Conversely, weekend admissions in urban areas showed higher rates of variceal hemorrhage compared to weekday admissions (2.0% vs. 1.9%, p < 0.001) (Table 2).

The use of hemodialysis for hepatorenal syndrome (HRS) was significantly lower in rural hospitals (0.1%) compared to urban hospitals (0.4%) (p < 0.001) (Table 2). There were no significant differences in hemodialysis rates or time to initiation of hemodialysis based on weekend versus weekday admission.

The prevalence of variceal hemorrhage was 2.6% in urban admissions and 2.5% in rural admissions (p < 0.001). For rural admissions, variceal hemorrhage occurred in 2.5% of weekday admissions and 2.7% of weekend admissions (p = 0.03). Endoscopic variceal band ligation (EVL) for variceal hemorrhage was performed in 1.5% of urban admissions and 0.9% of rural admissions (p < 0.001). Transjugular intrahepatic portosystemic shunt (TIPS) for variceal hemorrhage was performed in 0.2% of urban admissions and 0.1% of rural admissions (p = 0.02) (Table 2).

### Multivariable regression analysis

#### Mortality.

**Fig 3:**
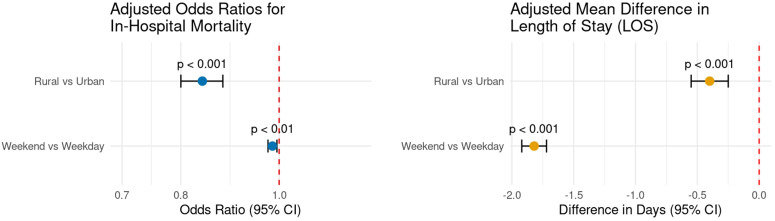
Adjusted Odds Ratios for In-hospital Mortality and Adjusted Mean Difference in LOS.

Results of the regression models of hospital admission outcomes by rurality and weekend admission are summarized in Table 3 and Fig 3. Weekend admissions were associated with a statistically significant but only modest reduction in the odds of in-hospital death compared to weekday admissions (OR 0.99, 95% CI: 0.975–0.998). Rural hospitalizations had lower odds of mortality compared to urban hospitalizations (OR 0.84, 95% CI: 0.80–0.86). The absolute mortality rate was 9.35% in urban hospitals versus 7.46% in rural hospitals, representing a 1.89% absolute risk reduction (95% CI: 1.68%−2.09%) for rural admissions. No significant interaction between rurality and weekend admission was observed in any of the models (all p > 0.05).

**Table 3:** Outcomes by rurality and weekend admission of hospitalizations for decompensated cirrhosis between 2016 and 2020.

Variable		Rural admission	95% CI	Weekend admission	95% CI	Interaction between rurality and weekend admission	95% CI
**In-hospital mortality** *Odds Ratio*		0.84	(0.81 - 0.86)	0.99	(0.98 - 1.00)	0.99	(0.95 - 1.04)
**Length of stay** (days) *Estimate*		−1.82	(−1.92 - −1.72)	−0.40	(−0.43 - −0.37)	−0.07	(−0.14 - 0.01)
**Procedures Received** *Odds Ratio*	Hemodialysis for HRS	0.40	(0.33 - 0.49)	0.95	(0.90 - 1.00)	0.98	(0.70 - 1.38)
	Paracentesis for SBP	0.54	(0.48 - 0.61)	0.94	(0.88 - 0.99)	0.99	(0.80 - 1.23)
	TIPS for VH	0.15	(0.09 - 0.26)	0.95	(0.86 - 1.06)	0.47	(0.13 - 0.76)
	All EGD for VH	0.45	(0.39 - 0.52)	1.05	(0.98 - 1.12)	0.85	(0.66 - 1.08)
	EGD with VBL for VH	0.49	(0.43 - 0.56)	1.05	(1.00 - 1.10)	0.94	(0.76 - 1.15)
**Days to Procedures Received** ^✝^ *Estimate*	Hemodialysis for HRS	−1.35	(−2.25 - −0.46)	−0.27	(−0.62 - −0.07)	0.03	(−1.15 - 1.21)
	Paracentesis for SBP	−0.23	(−0.49 - 0.03)	0.04	(−0.09 - 0.16)	−0.03	(−0.40 - 0.35)
	TIPS for VH	1.85	(−1.34 - 5.05)	0.29	(−0.05 - 0.63)	−2.01	(−5.28 - 1.27)
	All EGD for VH	−0.05	(−0.18 - 0.08)	0	(−0.06 - −0.06)	0.20	(−0.02 - 0.43)
	EGD with VBL for VH	−0.13	(−0.29 - 0.02)	−0.01	(−0.08 - 0.06)	0.19	(−0.08 - 0.46)

*Includes hospital admissions in which APR-DRG (all patient refined diagnosis related group) risk of mortality subclass was “extreme”.

✝Only among those who received these procedures.

Abbreviations: CI = confidence interval; EGD = upper endoscopy; HRS = hepatorenal syndrome; TIPS = transjugular intrahepatic portosystemic shunt; VBL = variceal band ligation; VH = variceal hemorrhage.

#### Length of stay (LOS).

Weekend admissions were associated with shorter LOS compared to weekday admissions (−0.40 days, 95% CI: −0.43 - −0.37). Rural hospitalizations were similarly associated with reduced LOS compared to urban hospitalizations (−1.82 days, 95% CI: −1.92 - −1.72). No interaction between rurality and weekend admission was observed for LOS.

#### Transfers to tertiary or acute care centers.

Among rural hospitals, the odds of transferring patients to tertiary care centers were 72% higher for those with the highest APR-DRG mortality scores (OR 1.72, 95% CI: 1.59–1.86). No significant differences in transfer rates were observed between weekend and weekday admissions (OR 0.96, 95% CI: 0.92–1.00).

Procedures received and Days to receive them were as follows:

**Hemodialysis:** Patients in rural hospitals had about half the odds of receiving hemodialysis than urban hospitals (OR 0.49, 95% CI: 0.33–0.49). No differences were observed in odds of hemodialysis based on weekend or weekday admission. There was also no significant interaction between rurality and weekend admission on the odds of receiving hemodialysis. Rural hospital admission was associated with fewer days to hemodialysis initiation compared to urban hospitals (−1.35 days, 95% CI: −2.59 - −0.11). There was no difference in days to hemodialysis for weekend vs. weekday admissions.

**Paracentesis:** The odds of receiving paracentesis were lower in rural hospitals than in urban hospitals (OR 0.54, 95% CI: 0.48–0.61). Weekend admissions were associated with slightly reduced odds of receiving paracentesis compared to weekday admissions (OR 0.94, 95% CI: 0.88–0.99). There was no significant difference in days to receive paracentesis in any of the models. There was no significant effect modification between rural and weekend admissions for patients who received paracentesis.

**UGE, VBL, and TIPS for Variceal Hemorrhage:** Rural hospitals had lower odds of performing UGE (OR 0.45, 95% CI: 0.39–0.52), VBL (OR 0.49, 95% CI: 0.43–0.56), and TIPS (OR 0.15, 95% CI: 0.09–0.26) than urban hospitals. Patients with the highest APR-DRG risk of mortality subclass had about half the odds of receiving UGE (OR 0.51, 95% CI: 0.44–0.58) but more than twice the odds of receiving TIPS (OR 2.88, 95% CI: 2.26–3.67). There was no difference in odds of receiving these procedures based on weekend or weekday admission. There was also no difference in days to receive these procedures in rural hospitals vs. urban hospitals or weekend vs. weekday admissions.

### Sensitivity analyses

We addressed potential survival bias by excluding all patients who were transferred to other acute care facilities. In this transfer-exclusive cohort, the overall rural survival advantage was slightly attenuated but remained statistically significant (aOR: 0.92; 95% CI: 0.88–0.95), with an absolute risk reduction of 1.58% (95% CI: 1.36%−1.79%) (Table 4). When further stratified by disease severity, rurality was associated with lower mortality among high-acuity patients (APR-DRG 3−4) both before and after excluding transfers (aOR: 0.82, 95% CI 0.79–0.85 and aOR 0.89; 95% CI: 0.86–0.93, respectively), representing an absolute risk reduction of 2.22% (95% CI: 1.96%−2.49 and 1.79% (95% CI: 1.52%−2.07%), respectively. Conversely, for patients with lower baseline mortality risk (APR-DRG 1−2) before and after excluding transfers, rural hospitalizations were associated with higher odds of mortality (aOR: 1.79; 95% CI 1.50–2.13 and aOR 1.86; 95% CI: 1.56–2.21, respectively), with an absolute risk increase of −0.59% (95% CI: −0.49% - −0.68%) and −0.64% (95% CI: −0.54% - −0.74%) (Table 4).

**Table 4:** Impact of Disease Severity and Patient Transfers on the Association Between Hospital Rurality and In-Hospital Mortality.

Patient Population	Urban Mortality (%)	Rural Mortality (%)	Absolute Risk Difference (95% CI)	Adjusted Odds Ratio (95% CI)
**Primary Analysis**				
Total Cohort (All)	9.35	7.46	1.89 (1.68, 2.09)	0.84 (0.80, 0.86)
**Sensitivity Analysis**				
Low-Mod Acuity (APR-DRG 1–2)	0.43	1.02	−0.59 (−0.68, −0.49)	1.79 (1.50, 2.14)
Severe-Extreme Acuity (APR-DRG 3–4)	11.94	9.72	2.22 (1.96, 2.49)	0.82 (0.78, 0.86)
Excluding Transfers (Overall)	9.61	8.04	1.58 (1.36, 1.79)	0.92 (0.88, 0.95)
Excluding Transfers (APR-DRG 1–2)	0.44	1.08	−0.64 (−0.74, −0.54)	1.86 (1.56, 2.21)
Excluding Transfers (APR-DRG 3–4)	12.31	10.52	1.79 (1.52, 2.07)	0.89 (0.86, 0.93)

Abbreviations: APR-DRG = all patient refined diagnosis related group.

Other sensitivity analyses for in-hospital mortality confirmed the stability of our primary estimates. When employing a stricter definition for cirrhosis to exclude potential misclassification, rural hospitalizations remained associated with lower mortality (OR: 0.81; 95% CI: 0.70–0.93). Similarly, excluding data from the year 2020 to account for the impact of the COVID-19 pandemic did not alter the direction or significance of the results (OR: 0.85; 95% CI: 0.80–0.91).

## Discussion

This study revisited the impact of rurality and weekend admission on outcomes in hospitalized patients with decompensated cirrhosis, using a large cohort from 2016 to 2020. One novel finding is the lower mortality rate in rural centers, coupled with a higher transfer rate, particularly for patients with severe clinical conditions. Additionally, we observed nuanced effects of weekend admissions, which may further inform resource allocation and care strategies in this high-risk population.

Contrary to prior findings suggesting worse outcomes for rural hospitalizations, our analysis demonstrated that rural hospitals were associated with slightly lower odds of in-hospital mortality compared to urban centers. This observation aligns with some recent studies suggesting that rural hospitals may have improved care protocols and smaller patient volumes, allowing more focused care in certain settings ([1]). However, our sensitivity analyses reveal a more nuanced clinical reality dependent on disease acuity. In high-acuity cases (APR-DRG 3–4), the survival benefit in rural hospitals was partially attenuated, but not eliminated, after excluding transferred patients. This indicates that while the higher rates of transfers from rural hospitals to tertiary centers reflect a “triage-first” approach that successfully shifts the highest-risk patients to higher levels of care, a residual “rural advantage” persists for those managed locally. In stark contrast, our finding of higher mortality for low-acuity patients (APR-DRG 1–2) in rural settings suggests a residual rural-urban disparity in the management of more stable cirrhosis, where urban centers may still benefit from more standardized specialty care and rapid-response infrastructure. Collectively, these findings suggest that the rural survival advantage in cirrhosis is driven by the efficient stabilization and triage of the most critically ill, rather than a universal superiority in care across all severity levels. Importantly, because the NIS attributes each death to the facility where it occurs, patients stabilized at rural hospitals and transferred to urban centers who subsequently die would have their mortality counted as an urban event, potentially inflating urban mortality and deflating rural mortality. Our sensitivity analyses excluding all transfers partially address this concern: the rural survival advantage was attenuated (aOR 0.92 vs. 0.84) but remained significant, suggesting that transfer-related attribution bias does not fully account for the observed difference.

Reduced staffing levels, fewer specialists, and delays in diagnostic or therapeutic interventions during weekends are commonly cited as contributing factors to higher mortality. In this study, weekend admissions were associated with a statistically significant but only modest reduction in mortality (OR 0.99, 95% CI: 0.975–0.998). Given the magnitude of this effect (roughly a 1% relative reduction in odds across nearly 12 million hospitalizations), this finding is best interpreted as the absence of a detrimental weekend effect in contemporary cirrhosis care, rather than as a true weekend survival advantage, likely reflecting the effectiveness of standardized protocols for managing acute decompensation.. Our data further revealed that patients with the highest APR-DRG risk of mortality were predominantly located in urban areas, with the majority admitted on weekdays. This pattern may reflect differences in care-seeking behavior, including patient perceptions of greater resource availability on the weekdays, or differences in provider referral practices whereby patients present to outpatient settings during the week and those who are more ill are subsequently advised to be admitted; these hypotheses warrant further study. Nonetheless, the lack of interaction between rurality and weekend admission suggests that this effect is independent of hospital location.

Shorter LOS for patients in rural hospitals may reflect differences in case complexity, availability of specialized services (e.g., intensive care or transplant evaluation) prompting earlier transfer to tertiary centers [10–12], or resource constraints contributing to earlier discharges in some cases [13,14]. Shorter LOS for weekend admissions across both urban and rural settings could reflect differences in discharge planning or procedural scheduling on weekends, which warrants further investigation [15].

Higher transfer rates from rural hospitals for critically ill patients highlights the importance of inter-hospital collaboration and highlights gaps in the capacity of rural hospitals to provide advanced care for critically ill patients [16]. Despite these higher transfer rates, rural hospitals continue to face systemic challenges, including staffing shortages and financial instability, which may impact long-term sustainability of care for cirrhosis patients [17].

Lower odds of receiving key procedures in rural hospitals is concerning given the critical role of these interventions in managing complications of decompensated cirrhosis. The lower rates of upper endoscopy and TIPS procedures in rural hospitals may reflect discrepancies in clinical guidelines or institutional policies, or limited availability of gastroenterology and interventional radiology specialists in rural healthcare settings [14]. The lower odds of performing TIPS in rural hospitals may contribute to worse outcomes for patients with variceal bleeding, underscoring the need for enhanced procedural capacity in these settings. Interestingly, while urban hospitals performed more procedures overall, rural hospitals demonstrated shorter times to initiate hemodialysis in cases when the procedure was performed, indicating possible differences among rural hospitals in the care provided for renal failure in cirrhotic patients. Modestly lower odds of paracentesis for weekend admissions may be attributable to reduced staffing, fewer on-site specialists, or lower procedural availability during weekends, a pattern that has been noted in other weekend-effect studies [18]. However, the lack of significant interaction between rurality and weekend admissions suggests these effects operate independently, rather than synergistically. Overall, however, the procedure analyses represent procedures performed at the index hospital only, therefore systemic factors such as transfer timing may confound the results (e.g., if patients are transferred out before a procedure is performed, or if the procedure is performed on an outpatient basis).

This study has several limitations. First, our data is derived from administrative datasets, which may lack granular clinical details, such as laboratory values or imaging findings, which could refine risk stratification. Although we adjusted for cirrhosis complications, comorbidities, and markers such as ascites, variceal hemorrhage, these variables may not fully capture all relevant clinical characteristics. We acknowledge that APR-DRG is an imperfect proxy for clinical severity; it is an administrative, partially outcome-dependent measure that does not incorporate liver-specific indices such as MELD or laboratory values (bilirubin, INR, creatinine, albumin), so residual confounding by unmeasured disease severity remains possible. Second, the NIS does not provide information on nonclinical determinants of healthcare access, such as social support, health literacy, transportation access, substance use history, or other socioeconomic factors which are critical for understanding patterns of access to advanced procedures like liver transplantation. Third, the deidentified nature of the NIS dataset precludes tracking rehospitalizations and outpatient procedures. As such, patients in rural hospitals might receive interventions (e.g., EGD) as outpatient care rather than during their hospitalization. Nonetheless, the conditions requiring urgent inpatient therapeutic procedures were thoroughly assessed, and these differences are unlikely to account fully for the observed differences. Notably, because the NIS captures only in-hospital mortality at the index facility and does not track outcomes across the full episode of care, post-transfer deaths are attributed to the receiving hospital rather than the originating institution. This design limitation may bias rural-urban mortality comparisons if transfer patterns differ systematically by hospital location, as demonstrated by the attenuation of the rural survival advantage in our transfer-exclusive sensitivity analysis. These include potential mislabeling or misclassification of clinical and demographic variables, as well as sampling bias among hospitals, despite the self-weighted design employed by the NIS. Finally, regional and hospital-specific differences in resource availability may have influenced our findings and require further exploration.

## Conclusions

This study examined the relationship between hospital rurality, weekend admission, and outcomes in patients with decompensated cirrhosis. After 2016, rural hospitals demonstrated slightly lower in-hospital mortality and shorter lengths of stay, but higher transfer rates and reduced access to essential procedures. Contrary to the “weekend effect,” weekend admissions were associated with a statistically significant but only modest reduction in mortality and shorter lengths of stay. Addressing these recent phenomena requires improving access to specialized procedures and strengthening rural hospitals’ capacity in treating clinically debilitated cirrhosis patients both during weekdays and weekends. Targeted investments in rural healthcare infrastructure, workforce development, and inter-hospital collaboration may help ensure equitable and high-quality care.

## Supporting information

S1 Table ICD-10-CM codes for cirrhosis and cirrhosis-related complications. (DOCX)

S2 Table Additional characteristics by rurality and weekend admission of hospital admissions for decompensated cirrhosis between 2016 and 2020. (DOCX)

## Abbreviations

APR-DRG: All Patient Refined Diagnosis Related Group
CLD: Chronic Liver Disease
COVID-19: Coronavirus Disease 2019
EGD: Esophagogastroduodenoscopy
HCUP: Healthcare Cost and Utilization Project
HRS: Hepatorenal Syndrome
ICD-10-CM: International Classification of Diseases, Tenth Revision, Clinical Modification
IQR: Interquartile Range
LOS: Length of Stay
MASLD: Metabolic Dysfunction-Associated Steatotic Liver Disease
NIS: National Inpatient Sample
OR: Odds Ratio
SBP: Spontaneous Bacterial Peritonitis
SD: Standard Deviation
STROBE: Strengthening the Reporting of Observational Studies in Epidemiology
TIPS: Transjugular Intrahepatic Portosystemic Shunt
UGE: Upper Gastrointestinal Endoscopy
UGIH: Upper Gastrointestinal Hemorrhage
VBL: Variceal Band Ligation
VH: Variceal Hemorrhage
