# Supplementary material for: Cirrhosis outcomes on rurality and weekend admissions revisited: A contemporary analysis of the national inpatient sample
Source: PLoS One. 2026 Jul 2;21(7):e0353178. doi: 10.1371/journal.pone.0353178 (PMC13327185; doi:10.1371/journal.pone.0353178)
Supplement: S1 Table — (DOCX) [file pone.0353178.s001.docx]

| **S1 Table.** ICD-10-CM codes for cirrhosis and cirrhosis-related complications. | |
| --- | --- |
| **Condition** | **ICD-10-CM codes** |
| **Cirrhosis** | K74, K70.3, K70.2 |
| **Complications** |  |
| Ascites | R18.8, K70.31, K70.11, K71.51 |
| Variceal hemorrhage | I85.01, I85.11 |
| Hepatic encephalopathy | G93.40, G93.41, G93.49, R40, K70.41, K71.11, K72.01, K72.11, K72.91, B19.0, B19.11, B19.21 |
| Hepatorenal syndrome | K76.7 |
| Hepatocellular carcinoma | C22.0, C22.8, C229 |
| Spontaneous bacterial peritonitis | K65.2 |
