# Supplementary material for: Cirrhosis outcomes on rurality and weekend admissions revisited: A contemporary analysis of the national inpatient sample
Source: PLoS One. 2026 Jul 2;21(7):e0353178. doi: 10.1371/journal.pone.0353178 (PMC13327185; doi:10.1371/journal.pone.0353178)
Supplement: S2 Table — (DOCX) [file pone.0353178.s002.docx]

| **S2 Table.** Additional characteristics by rurality and weekend admission of hospital admissions for decompensated cirrhosis between 2016 and 2020.   \| **Characteristics** \| **Total Urban  admissions** \| \| **Total Rural  admissions** \| \| **p-value** \| **Urban admissions** \| \| \| \| \| **Rural admissions** \| \| \| \| \| \| \| --- \| --- \| --- \| --- \| --- \| --- \| --- \| --- \| --- \| --- \| --- \| --- \| --- \| --- \| --- \| --- \| --- \| \| **Urban/Weekday  admissions** \| \| **Urban/Weekend  admissions** \| \| **p-value** \| **Rural/Weekday  admissions** \| \| **Rural/Weekend  admissions** \| \| \| **p-value** \| \| N/Mean \| (%)/SD \| N/Mean \| (%)/SD \| N/Mean \| (%)/SD \| N/Mean \| (%)/SD \| N/Mean \| (%)/SD \| \| N/Mean \| (%)/SD \| \| **Number of admissions** \| 10,973,098 \| 93% \| 872,125 \| 7.4% \|  \| 8,305,004 \| 76% \| 2,668,075 \| 24% \|  \| 660,630 \| 76% \| 211,495 \| \| 24% \|  \| \| **Demographics** \|  \|  \|  \|  \|  \|  \|  \|  \|  \|  \|  \|  \|  \| \|  \|  \| \| Age (years) \| 66.54 \| 15.49 \| 68.75 \| 14.57 \| <0.001 \| 66.53 \| 15.40 \| 66.57 \| 15.77 \| <0.001 \| 68.72 \| 14.48 \| 68.86 \| \| 14.83 \| 0.003 \| \| Sex (female) \| 5,265,109 \| 48% \| 443,370 \| 51% \| <0.001 \| 3,976,265 \| 48% \| 1,288,830 \| 48% \| <0.001 \| 334,615 \| 51% \| 108,755 \| \| 51% \| 0.006 \| \| Race \|  \|  \|  \|  \| <0.001 \|  \|  \|  \|  \| 0.002 \|  \|  \|  \| \|  \| 0.5 \| \| White \| 7,197,399 \| 66% \| 708,025 \| 81% \|  \| 5,452,414 \| 66% \| 1,744,980 \| 65% \|  \| 536,160 \| 81% \| 171,865 \| \| 81% \|  \| \| Black \| 1,584,555 \| 14% \| 73,505 \| 8.4% \|  \| 1,198,705 \| 14% \| 385,840 \| 14% \|  \| 55,560 \| 8.4% \| 17,945 \| \| 8.5% \|  \| \| Hispanic \| 1,238,380 \| 11% \| 73,505 \| 3.0% \|  \| 933,795 \| 11% \| 304,585 \| 11% \|  \| 19,910 \| 3.0% \| 6,085 \| \| 2.9% \|  \| \| Other \| 952,765 \| 8.7% \| 73,505 \| 7.4% \|  \| 720,090 \| 8.7% \| 232,670 \| 8.7% \|  \| 49,000 \| 7.4% \| 15,600 \| \| 7.4% \|  \| \| Insurance (Medicaid) \| 1,681,674 \| 15% \| 108,265 \| 12% \| <0.001 \| 1,259,610 \| 15% \| 422,060 \| 16% \| <0.001 \| 81,355 \| 12% \| 26,910 \| \| 13% \| 0.024 \| \| Region \|  \|  \|  \|  \| <0.001 \|  \|  \|  \|  \| <0.001 \|  \|  \|  \| \|  \| 0.151 \| \| Northeast \| 1,810,335 \| 16% \| 95,005 \| 11% \|  \| 1,380,250 \| 17% \| 430,085 \| 16% \|  \| 72,455 \| 11% \| 22,550 \| \| 11% \|  \| \| Midwest \| 2,312,996 \| 21% \| 236,115 \| 27% \|  \| 1,758,601 \| 21% \| 554,390 \| 21% \|  \| 178,990 \| 27% \| 57,125 \| \| 27% \|  \| \| South \| 4,404,610 \| 40% \| 443,506 \| 51% \|  \| 3,328,230 \| 40% \| 1,076,375 \| 40% \|  \| 335,871 \| 51% \| 107,635 \| \| 51% \|  \| \| West \| 2,445,156 \| 22% \| 97,500 \| 11% \|  \| 1,837,922 \| 22% \| 607,224 \| 23% \|  \| 73,315 \| 11% \| 24,185 \| \| 11% \|  \| \| **Etiology of Liver Disease** \|  \|  \|  \|  \|  \|  \|  \|  \|  \|  \|  \|  \|  \| \|  \|  \| \| Alcohol \| 1,613,694 \| 15% \| 111,815 \| 13% \| <0.001 \| 1,229,550 \| 15% \| 384,140 \| 14% \| <0.001 \| 86,160 \| 13% \| 25,655 \| \| 12% \| <0.001 \| \| MASLD \| 3,303,104 \| 30% \| 270,555 \| 31% \| <0.001 \| 2,529,655 \| 30% \| 773,450 \| 29% \| <0.001 \| 207,865 \| 31% \| 62,690 \| \| 30% \| <0.001 \| \| Hepatitis C \| 856,270 \| 7.8% \| 52,045 \| 6.0% \| <0.001 \| 652,255 \| 7.9% \| 204,015 \| 7.6% \| <0.001 \| 39,585 \| 6.0% \| 12,460 \| \| 5.9% \| 0.5 \| \| Hepatitis B \| 99,565 \| 0.9% \| 5,175 \| 0.6% \| <0.001 \| 75,430 \| 0.9% \| 24,135 \| 0.9% \| 0.8 \| 3,985 \| 0.6% \| 1,190 \| \| 60% \| 0.3 \| \| Autoimmune \| 52,585 \| 0.5% \| 3,005 \| 0.3% \| <0.001 \| 40,600 \| 0.5% \| 11,985 \| 0.4% \| <0.001 \| 2,340 \| 0.4% \| 665 \| \| 30% \| 0.2 \| \| Viral hepatitis \| 927,325 \| 8.5% \| 55,025 \| 6.3% \| <0.001 \| 706,345 \| 8.5% \| 220,980 \| 8.3% \| <0.001 \| 41,910 \| 6.3% \| 13,115 \| \| 6.2% \| 0.3 \| \| Other etiology \| 2,842,874 \| 26% \| 218,250 \| 25% \| <0.001 \| 2,180,710 \| 26% \| 662,160 \| 25% \| <0.001 \| 168,605 \| 26% \| 49,645 \| \| 23% \| <0.001 \| \| **Cirrhosis Complications** \|  \|  \|  \|  \|  \|  \|  \|  \|  \|  \|  \|  \|  \| \|  \|  \| \| Variceal hemorrhage \| 210,385 \| 1.9% \| 11,055 \| 1.3% \| <0.001 \| 156,065 \| 1.9% \| 54,320 \| 2.0% \| <0.001 \| 8,405 \| 1.3% \| 2,650 \| \| 1.3% \| 0.8 \| \| Ascites \| 2,426,200 \| 22% \| 156,250 \| 18% \| <0.001 \| 1,870,190 \| 23% \| 556,005 \| 21% \| <0.001 \| 121,300 \| 18% \| 34,950 \| \| 17% \| <0.001 \| \| SBP \| 163,735 \| 1.5% \| 10,935 \| 1.3% \| <0.001 \| 123,340 \| 1.5% \| 40,395 \| 1.5% \| 0.13 \| 8,405 \| 1.3% \| 2,530 \| \| 1.2% \| 0.2 \| \| Hepatorenal syndrome \| 210,700 \| 1.9% \| 12,880 \| 1.5% \| <0.001 \| 161,605 \| 1.9% \| 49,095 \| 1.8% \| <0.001 \| 10,005 \| 1.5% \| 2,875 \| \| 1.4% \| 0.025 \| \| **APR-DRG Risk of Mortality** \|  \|  \|  \|  \| <0.001 \|  \|  \|  \|  \| <0.001 \|  \|  \|  \| \|  \| <0.001 \| \| Moderate \| 2,472,174 \| 23% \| 226,160 \| 26% \|  \| 1,902,825 \| 23% \| 569,345 \| 21% \|  \| 174,075 \| 26% \| 52,085 \| \| 25% \|  \| \| Major \| 4,662,500 \| 42% \| 403,910 \| 46% \|  \| 3,543,240 \| 43% \| 1,119,255 \| 42% \|  \| 305,915 \| 46% \| 97,995 \| \| 46% \|  \| \| Extreme \| 3,838,424 \| 35% \| 242,055 \| 28% \|  \| 2,858,940 \| 34% \| 979,475 \| 3.7% \|  \| 180,640 \| 27% \| 61,415 \| \| 29% \|  \| \| **APR-DRG Severity of Illness** \|  \|  \|  \|  \| <0.001 \|  \|  \|  \|  \| <0.001 \|  \|  \|  \| \|  \| <0.001 \| \| Moderate \| 1,832,600 \| 17% \| 185,025 \| 21% \|  \| 1,410,250 \| 17% \| 422,350 \| 16% \|  \| 142,150 \| 22% \| 42,875 \| \| 20% \|  \| \| Major \| 4,981,419 \| 45% \| 426,675 \| 49% \|  \| 3,787,414 \| 46% \| 1,193,995 \| 45% \|  \| 323,870 \| 49% \| 102,805 \| \| 49% \|  \| \| Extreme \| 4,159,079 \| 38% \| 260,425 \| 30% \|  \| 3,107,340 \| 37% \| 1,051,730 \| 39% \|  \| 194,610 \| 29% \| 65,815 \| \| 31% \|  \|   Abbreviations: APR-DRG = all patient refined diagnosis related group; IQR = interquartile range; MASLD = metabolic dysfunction-associated steatotic liver disease; SBP = spontaneous bacterial peritonitis; SD = standard deviation. |
| --- | --- | --- | --- | --- | --- | --- | --- | --- | --- | --- | --- | --- | --- | --- | --- | --- | --- | --- | --- | --- | --- | --- | --- | --- | --- | --- | --- | --- | --- | --- | --- | --- | --- | --- | --- | --- | --- | --- | --- | --- | --- | --- | --- | --- | --- | --- | --- | --- | --- | --- | --- | --- | --- | --- | --- | --- | --- | --- | --- | --- | --- | --- | --- | --- | --- | --- | --- | --- | --- | --- | --- | --- | --- | --- | --- | --- | --- | --- | --- | --- | --- | --- | --- | --- | --- | --- | --- | --- | --- | --- | --- | --- | --- | --- | --- | --- | --- | --- | --- | --- | --- | --- | --- | --- | --- | --- | --- | --- | --- | --- | --- | --- | --- | --- | --- | --- | --- | --- | --- | --- | --- | --- | --- | --- | --- | --- | --- | --- | --- | --- | --- | --- | --- | --- | --- | --- | --- | --- | --- | --- | --- | --- | --- | --- | --- | --- | --- | --- | --- | --- | --- | --- | --- | --- | --- | --- | --- | --- | --- | --- | --- | --- | --- | --- | --- | --- | --- | --- | --- | --- | --- | --- | --- | --- | --- | --- | --- | --- | --- | --- | --- | --- | --- | --- | --- | --- | --- | --- | --- | --- | --- | --- | --- | --- | --- | --- | --- | --- | --- | --- | --- | --- | --- | --- | --- | --- | --- | --- | --- | --- | --- | --- | --- | --- | --- | --- | --- | --- | --- | --- | --- | --- | --- | --- | --- | --- | --- | --- | --- | --- | --- | --- | --- | --- | --- | --- | --- | --- | --- | --- | --- | --- | --- | --- | --- | --- | --- | --- | --- | --- | --- | --- | --- | --- | --- | --- | --- | --- | --- | --- | --- | --- | --- | --- | --- | --- | --- | --- | --- | --- | --- | --- | --- | --- | --- | --- | --- | --- | --- | --- | --- | --- | --- | --- | --- | --- | --- | --- | --- | --- | --- | --- | --- | --- | --- | --- | --- | --- | --- | --- | --- | --- | --- | --- | --- | --- | --- | --- | --- | --- | --- | --- | --- | --- | --- | --- | --- | --- | --- | --- | --- | --- | --- | --- | --- | --- | --- | --- | --- | --- | --- | --- | --- | --- | --- | --- | --- | --- | --- | --- | --- | --- | --- | --- | --- | --- | --- | --- | --- | --- | --- | --- | --- | --- | --- | --- | --- | --- | --- | --- | --- | --- | --- | --- | --- | --- | --- | --- | --- | --- | --- | --- | --- | --- | --- | --- | --- | --- | --- | --- | --- | --- | --- | --- | --- | --- | --- | --- | --- | --- | --- | --- | --- | --- | --- | --- | --- | --- | --- | --- | --- | --- | --- | --- | --- | --- | --- | --- | --- | --- | --- | --- | --- | --- | --- | --- | --- | --- | --- | --- | --- | --- | --- | --- | --- | --- | --- | --- | --- | --- | --- | --- | --- | --- | --- | --- | --- | --- | --- | --- | --- | --- | --- | --- | --- | --- | --- | --- | --- | --- | --- | --- | --- | --- | --- | --- | --- | --- | --- | --- | --- | --- | --- | --- | --- | --- | --- | --- | --- | --- | --- | --- | --- | --- | --- | --- | --- | --- | --- | --- | --- | --- | --- | --- | --- | --- | --- | --- | --- | --- | --- | --- | --- | --- | --- | --- | --- | --- | --- | --- | --- | --- | --- | --- | --- | --- | --- | --- | --- | --- | --- | --- | --- | --- | --- | --- | --- | --- | --- | --- | --- | --- | --- | --- | --- | --- | --- | --- | --- | --- | --- | --- | --- | --- | --- | --- | --- | --- | --- | --- | --- | --- | --- | --- | --- | --- | --- | --- | --- | --- | --- | --- | --- | --- | --- | --- | --- | --- | --- | --- | --- | --- | --- | --- | --- | --- | --- | --- | --- | --- | --- | --- | --- | --- | --- | --- | --- | --- | --- | --- | --- | --- | --- | --- | --- | --- | --- | --- | --- | --- | --- | --- | --- | --- | --- | --- | --- | --- | --- | --- | --- | --- | --- | --- | --- | --- | --- | --- | --- | --- | --- | --- | --- | --- | --- | --- | --- | --- | --- | --- | --- | --- | --- | --- | --- | --- | --- | --- | --- | --- | --- | --- | --- | --- | --- | --- | --- | --- | --- | --- | --- | --- | --- | --- | --- | --- | --- | --- | --- | --- | --- | --- | --- |
