# Supplementary material for: Cirrhosis outcomes on rurality and weekend admissions revisited: A contemporary analysis of the national inpatient sample
Source: PLoS One. 2026 Jul 2;21(7):e0353178. doi: 10.1371/journal.pone.0353178 (PMC13327185; doi:10.1371/journal.pone.0353178)
Supplement: Attachment — Submitted filename: Response to the reviewers.docx [file pone.0353178.s004.docx]

We thank the reviewers for their constructive critique of our manuscript. We believe that their thoughtful review has strengthened the quality of our manuscript.

Reviewers' comments:

Reviewer's Responses to Questions

**Comments to the Author**

1. Is the manuscript technically sound, and do the data support the conclusions?

The manuscript must describe a technically sound piece of scientific research with data that supports the conclusions. Experiments must have been conducted rigorously, with appropriate controls, replication, and sample sizes. The conclusions must be drawn appropriately based on the data presented.

Reviewer #1: Partly

Reviewer #2: Yes

2. Has the statistical analysis been performed appropriately and rigorously?

Reviewer #1: No

Reviewer #2: Yes

3. Have the authors made all data underlying the findings in their manuscript fully available?

The [PLOS Data policy](https://nam12.safelinks.protection.outlook.com/?url=http%3A%2F%2Ftrack.editorialmanager.com%2FCL0%2Fhttp%3A%252F%252Fwww.plosone.org%252Fstatic%252Fpolicies.action%2523sharing%2F1%2F010f019b2532fb25-b76b4ed6-164d-46e6-8417-23a870043edb-000000%2FkTR-L-Sn84xKKul1Pe8cnzzN_fT9v0OKjc71nDLuaG8%3D241&data=05%7C02%7Ctomohiro-tanaka%40uiowa.edu%7Cffc59b12aef8475b7f6108de4d37a2b0%7C1bc445959aba4fc3b8ec7b94a5586fdc%7C1%7C1%7C639033098526039358%7CUnknown%7CTWFpbGZsb3d8eyJFbXB0eU1hcGkiOnRydWUsIlYiOiIwLjAuMDAwMCIsIlAiOiJXaW4zMiIsIkFOIjoiTWFpbCIsIldUIjoyfQ%3D%3D%7C0%7C%7C%7C&sdata=cgP66DM0D8w1FzZipZnix7P%2Fnmcp%2FavRR7vUWkPL8X4%3D&reserved=0) requires authors to make all data underlying the findings described in their manuscript fully available without restriction, with rare exception (please refer to the Data Availability Statement in the manuscript PDF file). The data should be provided as part of the manuscript or its supporting information, or deposited to a public repository. For example, in addition to summary statistics, the data points behind means, medians and variance measures should be available. If there are restrictions on publicly sharing data—e.g. participant privacy or use of data from a third party—those must be specified.

Reviewer #1: Yes

Reviewer #2: Yes

4. Is the manuscript presented in an intelligible fashion and written in standard English?

PLOS ONE does not copyedit accepted manuscripts, so the language in submitted articles must be clear, correct, and unambiguous. Any typographical or grammatical errors should be corrected at revision, so please note any specific errors here.

Reviewer #1: Yes

Reviewer #2: Yes

5. Review Comments to the Author

Please use the space provided to explain your answers to the questions above. You may also include additional comments for the author, including concerns about dual publication, research ethics, or publication ethics. (Please upload your review as an attachment if it exceeds 20,000 characters)

Reviewer #1: The positive idea of this manuscript is that is clinically applicable. nevertheless, below some comments to improve your study

**Abstract**
Please clarify the lower mortality in rural hospitals. This may be driven by higher early transfer rates. Consider a sensitivity analysis excluding transferred patients or stratifying outcomes by transfer status
The authors should provide a clear description and justification of the ICD-10 codes used to identify decompensated cirrhosis (including ACLF and complications), and
expand on why rural hospitals performed fewer procedures despite similar time to procedure. Clarify whether severity adjustment was sufficient and discuss how reduced procedure use may affect guideline-based care and outcomes

**Response:** We thank reviewer 1 for this point. To directly address potential survival bias, we performed a sensitivity analysis excluding all patients transferred to other acute care facilities. In this transfer-exclusive cohort, the rural survival advantage remained statistically significant (aOR: 0.92; 95% CI: 0.88-0.95). Furthermore, when stratified by severity, the rural survival advantage remained robust for high-acuity patients (APR-DRG 3-4) even after excluding transfers (aOR: 0.89; 95% CI: 0.86-0.93). This suggests that while transfer patterns contribute to outcomes, they do not fully account for the observed mortality trends.

**Introduction Section**
Here, I have two points that require improvements:
• The introduction would benefit from briefly outlining the major causes of cirrhosis (e.g., MASLD/MASH, alcohol-associated liver disease, chronic viral hepatitis, autoimmune/metabolic causes). Providing etiologic context helps readers understand the burden and heterogeneity of decompensated cirrhosis in the U.S. population. suggested paper references:
• PMCID: PMC2922495, DOI: 10.1002/hep.23535
• PMID: 40745231, doi: 10.1007/s00418-025-02402-4
• PMID: 22131058 doi: 10.1007/s00418-011-0889-9

**Response:** We thank reviewer 1 for this point. We have addressed this and included a statement in the introduction regarding major causes of cirrhosis (with suggested reference papers) have been included (page 6, paragraph1).

The introduction jumps quickly between cirrhosis complications, weekend effect, and rural health disparities. Consider improving the logical flow and more clearly articulating the specific knowledge gap, i.e., that prior studies have examined weekend admissions and rurality separately, but the combined interaction and contemporary data post-2016 remain understudied. This will sharpen the rationale for the study.
I understand that probably due to word limit?

**Response:** Paragraph flow has been addressed, and the relevant knowledge gap has been emphasized (page 6, paragraphs 2-4).

Method
Case Definition and Cohort Selection Need Clearer Justification
As mentioned, the methods include patients with either a cirrhosis ICD-10 code or a complication code (ascites, HE, variceal bleed, …). This approach may misclassify acute conditions unrelated to cirrhosis (e.g., ascites from malignancy, encephalopathy from other causes). The authors have to provide stronger justification for this definition, clarify how non-cirrhotic etiologies were excluded, and consider adding sensitivity analyses using stricter cirrhosis codes.

**Response:** We have expanded the Methods section to provide a detailed justification for our ICD-10 selection. To address concerns regarding potential misclassification of non-cirrhotic conditions (e.g., malignancy-related ascites), we conducted a sensitivity analysis using a stricter definition requiring a primary diagnosis of cirrhosis. The results remained consistent with our primary findings, which are now detailed in the supplementary materials (page 9, paragraph 2) .


Statistical Modeling Description Is Dense and Lacks Clarity on Interaction Term
In view of statistical analysis, the description of the regression models is overly dense. It is unclear whether the interaction term was correctly specified (hospital rurality versus weekend admission) and whether multicollinearity or model diagnostics were assessed. I suggest to the author to consider simplifying the model description and explicitly stating how the interaction was tested and interpreted.

**Response:** We have simplified and clarified the statistical description to clarify that we tested the interaction between hospital rurality and weekend admission (page 8, paragraph 1).

Results

The overly large tables (Table 1 + 2) reduce readability. Indeed, both tables span two pages and require very small font size, making them difficult to read and interpret. Also, the key findings are buried in dense numerical text
The findings rely heavily on large tables and long paragraphs listing numbers, which makes it difficult for readers to quickly identify the most important differences and trends.
Convert the most clinically meaningful or statistically significant comparisons into graphs/figures (e.g., mortality rates, LOS, transfer rates, major complications). This is visually better and will improve clarity and streamline interpretation.

**Response:** We have condensed the primary tables and added Figure 1 (Clinical Outcomes, Hospital Costs, and Procedure Utilization based on location) and Figure 2 (mortality and LOS). These visualizations highlight clinically significant trends and improve readability.

Multivariable Regression Analysis
The regression findings are listed as numbers only, without explaining whether the differences are clinically meaningful (e.g., OR 0.99 for mortality, −0.40 days LOS). the author should add 1-2 sentences summarizing what these results mean clinically, for example:
the lower mortality and shorter LOS in rural hospitals may reflect differences in case mix, transfer patterns, or resource utilization…
The lack of interaction indicates that weekend admission does not modify the impact of rurality on outcomes.

**Response:** We have updated Table 4 to include absolute mortality rates and Absolute Risk Differences (RD) alongside the adjusted odds ratios. For example, in the primary cohort, rural hospitals were associated with a 1.89% absolute reduction in mortality (95% CI: 1.68%-2.09%). Conversely, in low-acuity cases (APR-DRG 1-2), rural mortality was 0.59% higher than urban mortality. Providing these absolute event rates allows for a clearer clinical interpretation of the magnitude of the effect across different severity subclasses (page 29, table 4).

Discussion
the author should take care of using causal language/ explanations in some paragraphs instead of scientific or clinical language (e.g., “critically ill patients may be aware…,” “premature discharges,” “prioritization of critical care on weekends”) without supporting evidence.
Unfortunately, this weakens the scientific rigor and may raise the concerns about speculation.
So, reframe these as hypotheses or possibilities, or explicitly state that these interpretations are speculative and require further study

**Response:** Causal language has been reframed to hypotheses/possibilities with emphasis on need for further study (pages 15, 16,17).

Large sections of the Discussion section restate / repeat the numeric findings (Result section) already presented (e.g., rural hospitals had lower mortality, higher transfers, fewer procedures), instead of discussing them! I shall advise the authors to condense the repeating/ redundant portions and shift emphasis to why these patterns may occur, how they compare with prior literature, and their implications for clinical practice and health policy

**Response:** Restating/repetition of results has been minimized in the discussion (pages 15,16,17).

Conflicting or paradoxes findings are acknowledged but not explored enough!
e.g., 1. rural hospitals have lower mortality despite fewer procedures
2. Weekend admissions have lower mortality despite lower procedure rates.
These inconsistencies should be addressed, otherwise the readers may question the internal coherence or confounding structure of the study.
Therefore, as a suggestion for the authors to add a targeted paragraph offering potential explanations (e.g., case-mix differences, earlier transfers, coding bias, residual severity confounding) and emphasize the need for caution when interpreting mortality as a quality metric.

**Response:** Thank you for this point. We have added text within the discussion section to clarify this (page 15, paragraph 1; page 17, paragraphs 1, 2).

Reviewer #2: The authors have performed a large, contemporary analysis of decompensated cirrhosis outcomes using the National Inpatient Sample, focusing on hospital rurality, weekend admissions, and their interaction. The topic is clinically relevant, the dataset is robust, and the analytic approach is generally appropriate. The finding of lower in-hospital mortality in rural hospitals and during weekend admissions is provocative and challenges prevailing assumptions, making this work potentially impactful.
However, several methodological clarifications, interpretive refinements, and additional sensitivity analyses are necessary to strengthen causal inference and avoid overinterpretation of administrative data.

Major Comments
1. Interpretation of Lower Rural Mortality
The conclusion that rural hospitals have lower in-hospital mortality requires more cautious framing.
---The higher transfer rate from rural hospitals, particularly among patients with extreme APR-DRG mortality risk, strongly suggests selection and survivor bias rather than superior rural care.
---Patients transferred out may die after transfer and are not captured as rural in-hospital deaths.
Suggestions:
---Explicitly state that the observed lower mortality likely reflects early triage and transfer practices, not improved outcomes.
---Consider a sensitivity analysis excluding transferred patients, or alternatively model transfer as a competing outcome.

**RESPONSE:** We have reframed our conclusions to acknowledge that lower rural mortality likely reflects early triage and transfer practices rather than superior care. To account for this, we performed a sensitivity analysis excluding all transferred patients, as suggested. While the rural survival advantage was attenuated in the transfer-exclusive cohort, it remained statistically significant (aOR: 0.92; 95% CI: 0.88-0.95), particularly among high-acuity patients (aOR: 0.89; 95% CI: 0.86-0.93) (page 14, paragraphs 2,3).

2. Use of APR-DRG Mortality Risk
APR-DRG mortality subclass is used as the primary severity adjustment, but this metric is hospital-assigned and partially outcome-dependent, which may introduce bias.
Suggestions:
---Clarify how APR-DRG is assigned temporally (at admission vs. discharge).
---Discuss limitations compared with liver-specific scores (e.g., MELD), which are unavailable in NIS.
---Consider stratified analyses limited to the “extreme” mortality subclass, where transfer behavior appears most influential.

**Response:** We have added a stratified analysis specifically focusing on the 'extreme' mortality subclass (APR-DRG 3-4). As requested, this allows for an isolated view of the patients most susceptible to transfer-related bias. Interestingly, the rural survival benefit persisted in this high-severity group (aOR: 0.82) even when including transfers, and remained significant (aOR: 0.89) when transfers were excluded, demonstrating the stability of the finding in the most critically ill patients (page 14, paragraphs 2, 3).

3. Weekend Effect Interpretation
The finding of lower mortality on weekend admissions (OR ~0.99) is statistically significant but clinically marginal.
---The effect size is very small and may reflect residual confounding, admission threshold differences, or coding variation.
---Statements suggesting prioritization of care on weekends are speculative.
Suggestions:
---Emphasize the minimal magnitude of effect.
---Reframe conclusions to note the absence of a harmful weekend effect rather than a protective one.
---Avoid implying causality or behavioral explanations without supporting data.

**Response:** The above changes have been made. Causal language has been reframed to hypotheses/possibilities with emphasis on need for further study (page 15, paragraph 2).

4. Procedure Utilization vs. Indication
Lower procedure rates (EGD, TIPS, paracentesis, dialysis) in rural hospitals may reflect:
---Limited availability
---Transfer prior to procedure
---Outpatient or post-transfer procedures not captured by NIS
Suggestions:
---Clarify that procedure analyses represent procedures performed at the index hospital only.
---Discuss how transfer timing may systematically reduce rural procedure counts.
---If possible, add models adjusting for transfer status.

**Response:** We have added Clarification regarding procedure analyses and possible confounding of transfer timing (page 17, paragraph 1)

5. COVID-19 Era Considerations
The study period includes 2020, when cirrhosis outcomes and hospital workflows changed substantially.
Suggestions:
---Add a sensitivity analysis excluding 2020, or
---Include year-by-year trends or a COVID indicator variable.

**Response:** Thank you for this point. We have included sensitivity analysis which excluded data from 2020. This interestingly did not alter the direction or significance of results (page 14, paragraph 3).

Minor Comments
---Briefly justify the use of the HCUP “dummy observation” method in the main text for readers unfamiliar with complex survey design.

**Response:** We have added a brief clarification in the Methods section justifying the use of the HCUP 'dummy observation' method to ensure the statistical integrity of our complex survey design (page 8, paragraph 2).

---Consider adding absolute event rates alongside odds ratios in Table 3 for clinical interpretability.

**Response:** Absolute mortality rates and risk differences were added to the analysis tables to provide a clearer clinical context alongside the adjusted odds ratios (Table 4).

---Expand the limitations section regarding unmeasured social determinants, outpatient care, and post-discharge mortality.

**Response:** Additional unmeasured social determinants have been added to the section. The inability to capture outpatient procedures and post-discharge mortality are included (page 17, paragraph 2).
