# Supplementary material for: Cirrhosis outcomes on rurality and weekend admissions revisited: A contemporary analysis of the national inpatient sample
Source: PLoS One. 2026 Jul 2;21(7):e0353178. doi: 10.1371/journal.pone.0353178 (PMC13327185; doi:10.1371/journal.pone.0353178)
Supplement: Attachment — Submitted filename: R2_Response to the reviewers_PLOSONE_Rural_WE_R2.docx [file pone.0353178.s005.docx]

6. Review Comments to the Author

Reviewer #1: Good progress, but a few issues remain:

Figure 1: Reconsider the color scheme. The current green-red combination may be difficult for readers with color blindness to distinguish. Consider selecting an alternative color palette that is more accessible.

RESPONSE: We thank the reviewer for this important suggestion regarding accessibility. We have replaced the green-red color palette in all figures with a colorblind-friendly blue-orange palette derived from the Okabe-Ito scheme. This change has been applied consistently across Figures 1 - 3.

Data presentation: authors are advised to convert more of the data currently presented in tables into graphical formats. Figures are often more effective for communicating key messages and improving readability. Increasing the number of figures while reducing the volume of numerical data in tables will make the manuscript clearer and more engaging for readers.

RESPONSE: We have added a new Figure 2 (and changed the name of the original Ffigure 2 to Figure 3); the new Fig 2 presents key clinical outcomes (in-hospital mortality, transfer rates, procedure utilization, and length of stay) stratified by hospital rurality. The corresponding tables have been retained as supplemental reference for readers who require exact numerical values.

I can agree to accept the current Response to the Reviewers; however, for the benefit of your manuscript and its future readers, I strongly encourage implementing these improvements. Well-designed figures can convey your message more effectively through visual interpretation rather than relying solely on numerical data.

Reviewer #2: The authors have done an excellent job addressing prior concerns, particularly regarding survival bias and methodological transparency. The addition of sensitivity analyses and stratified models significantly strengthens the validity of the findings. Please address following comments:

Minor Comments

1.The manuscript now incorporates APR-DRG appropriately and uses it for stratified analyses. However:
-APR-DRG remains an administrative, partially outcome-dependent measure.
-It does not capture liver-specific severity (e.g., MELD, lab values).
Suggestions:
-Explicitly acknowledge that APR-DRG is an imperfect proxy for clinical severity.
-Emphasize this limitation more clearly in the Discussion.

RESPONSE: We thank the reviewer for this important point and agree that APR-DRG is an imperfect proxy for clinical severity in cirrhosis. We have added the following sentence to the Limitations paragraph of the Discussion (page 23, lines 35-36 continuing to page 24, lines 1-3): *"In particular, although APR-DRG risk of mortality was used to adjust for and stratify by clinical severity, it remains an administrative, partially outcome-dependent classification and does not capture liver-specific severity metrics such as MELD score, serum bilirubin, INR, or creatinine; residual confounding by unmeasured disease severity therefore cannot be excluded."*

2.The authors have improved on weekend effect section, but interpretation can still be refined. The effect size (OR ~0.99) is statistically significant but clinically negligible. Current phrasing may still suggest a protective effect.
Suggestions:
-Reframe as: “No clinically meaningful weekend effect observed”
-Emphasize the minimal magnitude of effect.

Response: We thank the reviewer for this thoughtful suggestion. We agree that our prior phrasing could be read as implying a protective effect of weekend admission, which is not supported by the magnitude of the observed association. We have revised the language throughout the manuscript to more accurately reflect that, while the reduction in mortality odds reached statistical significance, its magnitude is modest and unlikely to be clinically meaningful. Specifically, we have made the following changes:

** Abstract (Results) --- page 4, line 16: revised to "Weekend admissions showed a statistically significant but only modest reduction in mortality odds (OR: 0.99; 95% CI: 0.975-0.998)."

** Abstract (Conclusions) --- page 4, lines 22-24: revised to "Using a national cohort of hospitalized patients with decompensated cirrhosis, we showed that rural hospitals exhibited lower in-hospital mortality, fewer procedures, and higher transfer rates, and that weekend admissions showed only a minimal, clinically insignificant reduction in mortality, irrespective of hospital rurality."

** Results section --- page 17, line 5 (continuing): revised to "Weekend admissions were associated with a statistically significant but only modest reduction in the odds of in-hospital death compared to weekday admissions (OR 0.99, 95% CI: 0.975-0.998)."

** Discussion --- page 22, lines 25-30: the opening of the weekend-effect paragraph has been rewritten to explicitly frame the finding as the absence of a detrimental weekend effect rather than a true weekend survival advantage: "In this study, weekend admissions were associated with a statistically significant but only modest reduction in mortality (OR 0.99, 95% CI: 0.975-0.998). Given the magnitude of this effect (roughly a 1% relative reduction in odds across nearly 12 million hospitalizations), this finding is best interpreted as the absence of a detrimental weekend effect in contemporary cirrhosis care, rather than as a true weekend survival advantage, likely reflecting the effectiveness of standardized protocols for managing acute decompensation."

** Conclusions --- page 24, lines 23-25: revised to "Contrary to the traditional 'weekend effect,' weekend admissions were associated with a statistically significant but only modest reduction in mortality and shorter lengths of stay."
