# Supplementary material for: Cirrhosis outcomes on rurality and weekend admissions revisited: A contemporary analysis of the national inpatient sample
Source: PLoS One. 2026 Jul 2;21(7):e0353178. doi: 10.1371/journal.pone.0353178 (PMC13327185; doi:10.1371/journal.pone.0353178)
Supplement: Attachment — Submitted filename: R3_Response to the reviewers_PLOSONE_Rural_WE_R3.docx [file pone.0353178.s006.docx]

**Response to the editorial suggestions:**

Additional Editor Comments :

Comment: One of the key findings of the manuscript is that patients initially managed at rural hospitals had lower odds of mortality, while transfer rates from rural hospitals appear to be substantial. The authors should clarify how mortality is defined in this study, specifically whether it reflects in-hospital mortality only or includes a defined post-discharge follow-up period. The observed survival advantage in rural settings requires further clarification, as it is possible that a proportion of adverse outcomes occurred after transfer to urban centers. If mortality is not tracked across the full episode of care, including post-transfer outcomes, this could potentially bias comparisons between rural and urban hospitals.

RESPONSE: We thank the editor for this important observation. Our primary outcome is in-hospital mortality at the admitting facility only. The NIS does not link records across facilities, so post-transfer deaths are attributed to the receiving institution and cannot be traced back to the originating admission. We agree this could bias rural–urban comparisons. To address this concern, we have made the following revisions:

1. Methods (page 7, 3^rd^ paragraph): Added an explicit definition clarifying that in-hospital mortality reflects death at the index facility and that post-transfer deaths are not linked back to the originating admission.

2. Discussion (page 22, 2^nd^ paragraph): Added text acknowledging that transfer-related attribution could inflate urban mortality and deflate rural mortality, and noting that our sensitivity analyses excluding transfers showed an attenuated but persistent rural advantage (aOR 0.92 vs. 0.84).

3. Limitations (page 24, limitation section [1^st^ paragraph]): Strengthened existing language to explicitly state that the NIS captures only index-facility mortality and that this design limitation may bias rural–urban comparisons when transfer patterns differ systematically
