# Supplementary material for: Cirrhosis outcomes on rurality and weekend admissions revisited: A contemporary analysis of the national inpatient sample
Source: PLoS One. 2026 Jul 2;21(7):e0353178. doi: 10.1371/journal.pone.0353178 (PMC13327185; doi:10.1371/journal.pone.0353178)
Supplement: Peer review history

# Peer review: Decision Letter 0

**DOI:** 10.1371/journal.pone.0353178.r001

**Authors:** Usama Waqar

15 Dec 2025

PONE-D-25-55717

Cirrhosis Outcomes on Rurality and Weekend Admissions Revisited: A Contemporary Analysis of the National Inpatient Sample

PLOS One

Dear Dr. Tanaka,

Thank you for submitting your manuscript to PLOS ONE. After careful consideration, we feel that it has merit but does not fully meet PLOS ONE’s publication criteria as it currently stands. Therefore, we invite you to submit a revised version of the manuscript that addresses the points raised during the review process.

Please refer to the comments from reviewers below.

Please submit your revised manuscript by Jan 29 2026 11:59PM. If you will need more time than this to complete your revisions, please reply to this message or contact the journal office at plosone@plos.org. When you're ready to submit your revision, log on to https://www.editorialmanager.com/pone/ and select the 'Submissions Needing Revision' folder to locate your manuscript file.

Please include the following items when submitting your revised manuscript:

- A letter that responds to each point raised by the academic editor and reviewer(s). You should upload this letter as a separate file labeled 'Response to Reviewers'.
- A marked-up copy of your manuscript that highlights changes made to the original version. You should upload this as a separate file labeled 'Revised Manuscript with Track Changes'.
- An unmarked version of your revised paper without tracked changes. You should upload this as a separate file labeled 'Manuscript'.

If you would like to make changes to your financial disclosure, please include your updated statement in your cover letter. Guidelines for resubmitting your figure files are available below the reviewer comments at the end of this letter.

If applicable, we recommend that you deposit your laboratory protocols in protocols.io to enhance the reproducibility of your results. Protocols.io assigns your protocol its own identifier (DOI) so that it can be cited independently in the future. For instructions see: https://journals.plos.org/plosone/s/submission-guidelines#loc-laboratory-protocols. Additionally, PLOS ONE offers an option for publishing peer-reviewed Lab Protocol articles, which describe protocols hosted on protocols.io. Read more information on sharing protocols at https://plos.org/protocols?utm_medium=editorial-email&utm_source=authorletters&utm_campaign=protocols.

We look forward to receiving your revised manuscript.

Kind regards,

Usama Waqar, M.B.B.S

Academic Editor

PLOS One

Journal Requirements:

When submitting your revision, we need you to address these additional requirements.

1. Please ensure that your manuscript meets PLOS ONE's style requirements, including those for file naming. The PLOS ONE style templates can be found at

https://journals.plos.org/plosone/s/file?id=wjVg/PLOSOne_formatting_sample_main_body.pdf and

https://journals.plos.org/plosone/s/file?id=ba62/PLOSOne_formatting_sample_title_authors_affiliations.pdf

2. PLOS requires an ORCID iD for the corresponding author in Editorial Manager on papers submitted after December 6th, 2016. Please ensure that you have an ORCID iD and that it is validated in Editorial Manager. To do this, go to ‘Update my Information’ (in the upper left-hand corner of the main menu), and click on the Fetch/Validate link next to the ORCID field. This will take you to the ORCID site and allow you to create a new iD or authenticate a pre-existing iD in Editorial Manager.

3. Thank you for stating the following financial disclosure:

“This study was funded by the divisional fund at the University of Iowa Division of Gastroenterology and Hepatology (the Bruce L. Miller & M. Joyce Miller Memorial & Visiting Professorship).”

Please state what role the funders took in the study.  If the funders had no role, please state: "The funders had no role in study design, data collection and analysis, decision to publish, or preparation of the manuscript."

If this statement is not correct you must amend it as needed.

Please include this amended Role of Funder statement in your cover letter; we will change the online submission form on your behalf.

4. We note that the grant information you provided in the ‘Funding Information’ and ‘Financial Disclosure’ sections do not match.

When you resubmit, please ensure that you provide the correct grant numbers for the awards you received for your study in the ‘Funding Information’ section.

5. Please include your full ethics statement in the ‘Methods’ section of your manuscript file. In your statement, please include the full name of the IRB or ethics committee who approved or waived your study, as well as whether or not you obtained informed written or verbal consent. If consent was waived for your study, please include this information in your statement as well.

6. If the reviewer comments include a recommendation to cite specific previously published works, please review and evaluate these publications to determine whether they are relevant and should be cited. There is no requirement to cite these works unless the editor has indicated otherwise.

[Note: HTML markup is below. Please do not edit.]

Reviewers' comments:

Reviewer's Responses to Questions

**Comments to the Author**

1. Is the manuscript technically sound, and do the data support the conclusions?

The manuscript must describe a technically sound piece of scientific research with data that supports the conclusions. Experiments must have been conducted rigorously, with appropriate controls, replication, and sample sizes. The conclusions must be drawn appropriately based on the data presented.

Reviewer #1: Partly

Reviewer #2: Yes

**********

2. Has the statistical analysis been performed appropriately and rigorously?

Reviewer #1: No

Reviewer #2: Yes

**********

3. Have the authors made all data underlying the findings in their manuscript fully available?

The PLOS Data policy requires authors to make all data underlying the findings described in their manuscript fully available without restriction, with rare exception (please refer to the Data Availability Statement in the manuscript PDF file). The data should be provided as part of the manuscript or its supporting information, or deposited to a public repository. For example, in addition to summary statistics, the data points behind means, medians and variance measures should be available. If there are restrictions on publicly sharing data—e.g. participant privacy or use of data from a third party—those must be specified.

Reviewer #1: Yes

Reviewer #2: Yes

**********

4. Is the manuscript presented in an intelligible fashion and written in standard English?

PLOS ONE does not copyedit accepted manuscripts, so the language in submitted articles must be clear, correct, and unambiguous. Any typographical or grammatical errors should be corrected at revision, so please note any specific errors here.

Reviewer #1: Yes

Reviewer #2: Yes

**********

5. Review Comments to the Author

Please use the space provided to explain your answers to the questions above. You may also include additional comments for the author, including concerns about dual publication, research ethics, or publication ethics. (Please upload your review as an attachment if it exceeds 20,000 characters)

Reviewer #1: The positive idea of this manuscript is that is clinically applicable. nevertheless, below some comments to improve your study

Abstractt

Please clarify the lower mortality in rural hospitals. This may be driven by higher early transfer rates. Consider a sensitivity analysis excluding transferred patients or stratifying outcomes by transfer status

The authors should provide a clear description and justification of the ICD-10 codes used to identify decompensated cirrhosis (including ACLF and complications), and

expand on why rural hospitals performed fewer procedures despite similar time to procedure. Clarify whether severity adjustment was sufficient and discuss how reduced procedure use may affect guideline-based care and outcomes

Introduction Section

Here, I have two points that require improvements:

• The introduction would benefit from briefly outlining the major causes of cirrhosis (e.g., MASLD/MASH, alcohol-associated liver disease, chronic viral hepatitis, autoimmune/metabolic causes). Providing etiologic context helps readers understand the burden and heterogeneity of decompensated cirrhosis in the U.S. population. suggested paper references:

• PMCID: PMC2922495, DOI: 10.1002/hep.23535

• PMID: 40745231, doi: 10.1007/s00418-025-02402-4

• PMID: 22131058 doi: 10.1007/s00418-011-0889-9

The introduction jumps quickly between cirrhosis complications, weekend effect, and rural health disparities. Consider improving the logical flow and more clearly articulating the specific knowledge gap, i.e., that prior studies have examined weekend admissions and rurality separately, but the combined interaction and contemporary data post-2016 remain understudied. This will sharpen the rationale for the study.

I understand that probably due to word limit?

Method

Case Definition and Cohort Selection Need Clearer Justification

As mentioned, the methods include patients with either a cirrhosis ICD-10 code or a complication code (ascites, HE, variceal bleed, …). This approach may misclassify acute conditions unrelated to cirrhosis (e.g., ascites from malignancy, encephalopathy from other causes). The authors have to provide stronger justification for this definition, clarify how non-cirrhotic etiologies were excluded, and consider adding sensitivity analyses using stricter cirrhosis codes.

Statistical Modeling Description Is Dense and Lacks Clarity on Interaction Term

In view of statistical analysis, the description of the regression models is overly dense. It is unclear whether the interaction term was correctly specified (hospital rurality versus weekend admission) and whether multicollinearity or model diagnostics were assessed. I suggest to the author to consider simplifying the model description and explicitly stating how the interaction was tested and interpreted.

Results

The overly large tables (Table 1 + 2) reduce readability. Indeed, both tables span two pages and require very small font size, making them difficult to read and interpret. Also, the key findings are buried in dense numerical text

The findings rely heavily on large tables and long paragraphs listing numbers, which makes it difficult for readers to quickly identify the most important differences and trends.

Convert the most clinically meaningful or statistically significant comparisons into graphs/figures (e.g., mortality rates, LOS, transfer rates, major complications). This is visually better and will improve clarity and streamline interpretation.

Multivariable Regression Analysis

The regression findings are listed as numbers only, without explaining whether the differences are clinically meaningful (e.g., OR 0.99 for mortality, −0.40 days LOS). the author should add 1–2 sentences summarizing what these results mean clinically, for example:

he lower mortality and shorter LOS in rural hospitals may reflect differences in case mix, transfer patterns, or resource utilization…

The lack of interaction indicates that weekend admission does not modify the impact of rurality on outcomes.

Discussion

the author should take care of using causal language/ explanations in some paragraphs instead of scientific or clinical language (e.g., “critically ill patients may be aware…,” “premature discharges,” “prioritization of critical care on weekends”) without supporting evidence.

Unfortunately, this weakens the scientific rigor and may raise the concerns about speculation.

So, reframe these as hypotheses or possibilities, or explicitly state that these interpretations are speculative and require further study

Large sections of the Discussion section restate / repeat the numeric findings (Result section) already presented (e.g., rural hospitals had lower mortality, higher transfers, fewer procedures), instead of discussing them! I shall advise the authors to condense the repeating/ redundant portions and shift emphasis to why these patterns may occur, how they compare with prior literature, and their implications for clinical practice and health policy

Conflicting or paradoxes findings are acknowledged but not explored enough!

e.g., 1. rural hospitals have lower mortality despite fewer procedures

2. Weekend admissions have lower mortality despite lower procedure rates.

These inconsistencies should be addressed, otherwise the readers may question the internal coherence or confounding structure of the study.

Therefore, as a suggestion for the authors to add a targeted paragraph offering potential explanations (e.g., case-mix differences, earlier transfers, coding bias, residual severity confounding) and emphasize the need for caution when interpreting mortality as a quality metric.

Reviewer #2: The authors have performed a large, contemporary analysis of decompensated cirrhosis outcomes using the National Inpatient Sample, focusing on hospital rurality, weekend admissions, and their interaction. The topic is clinically relevant, the dataset is robust, and the analytic approach is generally appropriate. The finding of lower in-hospital mortality in rural hospitals and during weekend admissions is provocative and challenges prevailing assumptions, making this work potentially impactful.

However, several methodological clarifications, interpretive refinements, and additional sensitivity analyses are necessary to strengthen causal inference and avoid overinterpretation of administrative data.

Major Comments

1. Interpretation of Lower Rural Mortality

The conclusion that rural hospitals have lower in-hospital mortality requires more cautious framing.

• The higher transfer rate from rural hospitals, particularly among patients with extreme APR-DRG mortality risk, strongly suggests selection and survivor bias rather than superior rural care.

• Patients transferred out may die after transfer and are not captured as rural in-hospital deaths.

Suggestions:

• Explicitly state that the observed lower mortality likely reflects early triage and transfer practices, not improved outcomes.

• Consider a sensitivity analysis excluding transferred patients, or alternatively model transfer as a competing outcome.

2. Use of APR-DRG Mortality Risk

APR-DRG mortality subclass is used as the primary severity adjustment, but this metric is hospital-assigned and partially outcome-dependent, which may introduce bias.

Suggestions:

• Clarify how APR-DRG is assigned temporally (at admission vs. discharge).

• Discuss limitations compared with liver-specific scores (e.g., MELD), which are unavailable in NIS.

• Consider stratified analyses limited to the “extreme” mortality subclass, where transfer behavior appears most influential.

3. Weekend Effect Interpretation

The finding of lower mortality on weekend admissions (OR ~0.99) is statistically significant but clinically marginal.

• The effect size is very small and may reflect residual confounding, admission threshold differences, or coding variation.

• Statements suggesting prioritization of care on weekends are speculative.

Suggestions:

• Emphasize the minimal magnitude of effect.

• Reframe conclusions to note the absence of a harmful weekend effect rather than a protective one.

• Avoid implying causality or behavioral explanations without supporting data.

4. Procedure Utilization vs. Indication

Lower procedure rates (EGD, TIPS, paracentesis, dialysis) in rural hospitals may reflect:

• Limited availability

• Transfer prior to procedure

• Outpatient or post-transfer procedures not captured by NIS

Suggestions:

• Clarify that procedure analyses represent procedures performed at the index hospital only.

• Discuss how transfer timing may systematically reduce rural procedure counts.

• If possible, add models adjusting for transfer status.

5. COVID-19 Era Considerations

The study period includes 2020, when cirrhosis outcomes and hospital workflows changed substantially.

Suggestions:

• Add a sensitivity analysis excluding 2020, or

• Include year-by-year trends or a COVID indicator variable.

Minor Comments

• Briefly justify the use of the HCUP “dummy observation” method in the main text for readers unfamiliar with complex survey design.

• Consider adding absolute event rates alongside odds ratios in Table 3 for clinical interpretability.

• Expand the limitations section regarding unmeasured social determinants, outpatient care, and post-discharge mortality.

**********

6. PLOS authors have the option to publish the peer review history of their article (what does this mean?). If published, this will include your full peer review and any attached files.

If you choose “no”, your identity will remain anonymous but your review may still be made public.

**Do you want your identity to be public for this peer review?**  For information about this choice, including consent withdrawal, please see our Privacy Policy.

Reviewer #1: No

Reviewer #2: No

**********

[NOTE: If reviewer comments were submitted as an attachment file, they will be attached to this email and accessible via the submission site. Please log into your account, locate the manuscript record, and check for the action link "View Attachments". If this link does not appear, there are no attachment files.]

To ensure your figures meet our technical requirements, please review our figure guidelines: https://journals.plos.org/plosone/s/figures

You may also use PLOS’s free figure tool, NAAS, to help you prepare publication quality figures: https://journals.plos.org/plosone/s/figures#loc-tools-for-figure-preparation.

NAAS will assess whether your figures meet our technical requirements by comparing each figure against our figure specifications.

# Author comment: Author response to Decision Letter 1

**DOI:** 10.1371/journal.pone.0353178.r002

13 Feb 2026

We thank the reviewers for their constructive critique of our manuscript. We believe that their thoughtful review has strengthened the quality of our manuscript.

Attachment Submitted filename: Response to the reviewers.docx

# Peer review: Decision Letter 1

**DOI:** 10.1371/journal.pone.0353178.r003

**Authors:** Usama Waqar, Usama Waqar

13 Apr 2026

PONE-D-25-55717R1

Cirrhosis Outcomes on Rurality and Weekend Admissions Revisited: A Contemporary Analysis of the National Inpatient Sample

PLOS One

Dear Dr. Tanaka,

Thank you for submitting your manuscript to PLOS ONE. After careful consideration, we feel that it has merit but does not fully meet PLOS ONE’s publication criteria as it currently stands. Therefore, we invite you to submit a revised version of the manuscript that addresses the points raised during the review process.

Please find reviewer comments below.

Please submit your revised manuscript by May 28 2026 11:59PM. If you will need more time than this to complete your revisions, please reply to this message or contact the journal office at plosone@plos.org. When you're ready to submit your revision, log on to https://www.editorialmanager.com/pone/ and select the 'Submissions Needing Revision' folder to locate your manuscript file.

Please include the following items when submitting your revised manuscript:

- A letter that responds to each point raised by the academic editor and reviewer(s). You should upload this letter as a separate file labeled 'Response to Reviewers'.
- A marked-up copy of your manuscript that highlights changes made to the original version. You should upload this as a separate file labeled 'Revised Manuscript with Track Changes'.
- An unmarked version of your revised paper without tracked changes. You should upload this as a separate file labeled 'Manuscript'.

If you would like to make changes to your financial disclosure, please include your updated statement in your cover letter. Guidelines for resubmitting your figure files are available below the reviewer comments at the end of this letter.

If applicable, we recommend that you deposit your laboratory protocols in protocols.io to enhance the reproducibility of your results. Protocols.io assigns your protocol its own identifier (DOI) so that it can be cited independently in the future. For instructions see: https://journals.plos.org/plosone/s/submission-guidelines#loc-laboratory-protocols. Additionally, PLOS ONE offers an option for publishing peer-reviewed Lab Protocol articles, which describe protocols hosted on protocols.io. Read more information on sharing protocols at https://plos.org/protocols?utm_medium=editorial-email&utm_source=authorletters&utm_campaign=protocols.

As the corresponding author, your ORCID iD is verified in the submission system and will appear in the published article. PLOS supports the use of ORCID, and we encourage all coauthors to register for an ORCID iD and use it as well. Please encourage your coauthors to verify their ORCID iD within the submission system before final acceptance, as unverified ORCID iDs will not appear in the published article. *Only*  the individual author can complete the verification step; PLOS staff *cannot*  verify ORCID iDs on behalf of authors.

We look forward to receiving your revised manuscript.

Kind regards,

Usama Waqar, M.B.B.S

Academic Editor

PLOS One

**Journal Requirements:**

1. If the reviewer comments include a recommendation to cite specific previously published works, please review and evaluate these publications to determine whether they are relevant and should be cited. There is no requirement to cite these works unless the editor has indicated otherwise.

2. Please review your reference list to ensure that it is complete and correct. If you have cited papers that have been retracted, please include the rationale for doing so in the manuscript text, or remove these references and replace them with relevant current references. Any changes to the reference list should be mentioned in the rebuttal letter that accompanies your revised manuscript. If you need to cite a retracted article, indicate the article’s retracted status in the References list and also include a citation and full reference for the retraction notice.

[Note: HTML markup is below. Please do not edit.]

Reviewers' comments:

Reviewer's Responses to Questions

**Comments to the Author**

1. If the authors have adequately addressed your comments raised in a previous round of review and you feel that this manuscript is now acceptable for publication, you may indicate that here to bypass the “Comments to the Author” section, enter your conflict of interest statement in the “Confidential to Editor” section, and submit your "Accept" recommendation.

Reviewer #1: All comments have been addressed

Reviewer #2: All comments have been addressed

**********

2. Is the manuscript technically sound, and do the data support the conclusions?

The manuscript must describe a technically sound piece of scientific research with data that supports the conclusions. Experiments must have been conducted rigorously, with appropriate controls, replication, and sample sizes. The conclusions must be drawn appropriately based on the data presented.

Reviewer #1: Yes

Reviewer #2: Yes

**********

3. Has the statistical analysis been performed appropriately and rigorously?

Reviewer #1: Yes

Reviewer #2: Yes

**********

4. Have the authors made all data underlying the findings in their manuscript fully available?

The PLOS Data policy requires authors to make all data underlying the findings described in their manuscript fully available without restriction, with rare exception (please refer to the Data Availability Statement in the manuscript PDF file). The data should be provided as part of the manuscript or its supporting information, or deposited to a public repository. For example, in addition to summary statistics, the data points behind means, medians and variance measures should be available. If there are restrictions on publicly sharing data—e.g. participant privacy or use of data from a third party—those must be specified.

Reviewer #1: Yes

Reviewer #2: Yes

**********

5. Is the manuscript presented in an intelligible fashion and written in standard English?

PLOS ONE does not copyedit accepted manuscripts, so the language in submitted articles must be clear, correct, and unambiguous. Any typographical or grammatical errors should be corrected at revision, so please note any specific errors here.

Reviewer #1: Yes

Reviewer #2: Yes

**********

6. Review Comments to the Author

Please use the space provided to explain your answers to the questions above. You may also include additional comments for the author, including concerns about dual publication, research ethics, or publication ethics. (Please upload your review as an attachment if it exceeds 20,000 characters)

Reviewer #1: Good progress, but a few issues remain:

Figure 1: Reconsider the color scheme. The current green–red combination may be difficult for readers with color blindness to distinguish. Consider selecting an alternative color palette that is more accessible.

Data presentation: authors are advised to convert more of the data currently presented in tables into graphical formats. Figures are often more effective for communicating key messages and improving readability. Increasing the number of figures while reducing the volume of numerical data in tables will make the manuscript clearer and more engaging for readers.

I can agree to accept the current Response to the Reviewers; however, for the benefit of your manuscript and its future readers, I strongly encourage implementing these improvements. Well-designed figures can convey your message more effectively through visual interpretation rather than relying solely on numerical data.

Reviewer #2: The authors have done an excellent job addressing prior concerns, particularly regarding survival bias and methodological transparency. The addition of sensitivity analyses and stratified models significantly strengthens the validity of the findings. Please address following comments:

Minor Comments

1.The manuscript now incorporates APR-DRG appropriately and uses it for stratified analyses. However:

• APR-DRG remains an administrative, partially outcome-dependent measure.

• It does not capture liver-specific severity (e.g., MELD, lab values).

Suggestions:

• Explicitly acknowledge that APR-DRG is an imperfect proxy for clinical severity.

• Emphasize this limitation more clearly in the Discussion.

2.The authors have improved on weekend effect section, but interpretation can still be refined. The effect size (OR ~0.99) is statistically significant but clinically negligible. Current phrasing may still suggest a protective effect.

Suggestions:

• Reframe as: “No clinically meaningful weekend effect observed”

• Emphasize the minimal magnitude of effect.

**********

7. PLOS authors have the option to publish the peer review history of their article (what does this mean?). If published, this will include your full peer review and any attached files.

If you choose “no”, your identity will remain anonymous but your review may still be made public.

**Do you want your identity to be public for this peer review?**  For information about this choice, including consent withdrawal, please see our Privacy Policy.

Reviewer #1: No

Reviewer #2: No

**********

[NOTE: If reviewer comments were submitted as an attachment file, they will be attached to this email and accessible via the submission site. Please log into your account, locate the manuscript record, and check for the action link "View Attachments". If this link does not appear, there are no attachment files.]

To ensure your figures meet our technical requirements, please review our figure guidelines: https://journals.plos.org/plosone/s/figures

You may also use PLOS’s free figure tool, NAAS, to help you prepare publication quality figures: https://journals.plos.org/plosone/s/figures#loc-tools-for-figure-preparation.

NAAS will assess whether your figures meet our technical requirements by comparing each figure against our figure specifications.

# Author comment: Author response to Decision Letter 2

**DOI:** 10.1371/journal.pone.0353178.r004

16 Apr 2026

6. Review Comments to the Author

Reviewer #1: Good progress, but a few issues remain:

Figure 1: Reconsider the color scheme. The current green-red combination may be difficult for readers with color blindness to distinguish. Consider selecting an alternative color palette that is more accessible.

RESPONSE: We thank the reviewer for this important suggestion regarding accessibility. We have replaced the green-red color palette in all figures with a colorblind-friendly blue-orange palette derived from the Okabe-Ito scheme. This change has been applied consistently across Figures 1 - 3.

Data presentation: authors are advised to convert more of the data currently presented in tables into graphical formats. Figures are often more effective for communicating key messages and improving readability. Increasing the number of figures while reducing the volume of numerical data in tables will make the manuscript clearer and more engaging for readers.

RESPONSE: We have added a new Figure 2 (and changed the name of the original Ffigure 2 to Figure 3); the new Fig 2 presents key clinical outcomes (in-hospital mortality, transfer rates, procedure utilization, and length of stay) stratified by hospital rurality. The corresponding tables have been retained as supplemental reference for readers who require exact numerical values.

Reviewer #2:

1.The manuscript now incorporates APR-DRG appropriately and uses it for stratified analyses. However:

-APR-DRG remains an administrative, partially outcome-dependent measure.

-It does not capture liver-specific severity (e.g., MELD, lab values).

Suggestions:

-Explicitly acknowledge that APR-DRG is an imperfect proxy for clinical severity.

-Emphasize this limitation more clearly in the Discussion.

RESPONSE: We thank the reviewer for this important point and agree that APR-DRG is an imperfect proxy for clinical severity in cirrhosis. We have added the following sentence to the Limitations paragraph of the Discussion (page 23, lines 35-36 continuing to page 24, lines 1-3): "In particular, although APR-DRG risk of mortality was used to adjust for and stratify by clinical severity, it remains an administrative, partially outcome-dependent classification and does not capture liver-specific severity metrics such as MELD score, serum bilirubin, INR, or creatinine; residual confounding by unmeasured disease severity therefore cannot be excluded."

2.The authors have improved on weekend effect section, but interpretation can still be refined. The effect size (OR ~0.99) is statistically significant but clinically negligible. Current phrasing may still suggest a protective effect.

Suggestions:

-Reframe as: “No clinically meaningful weekend effect observed”

-Emphasize the minimal magnitude of effect.

Response: We thank the reviewer for this thoughtful suggestion. We agree that our prior phrasing could be read as implying a protective effect of weekend admission, which is not supported by the magnitude of the observed association. We have revised the language throughout the manuscript to more accurately reflect that, while the reduction in mortality odds reached statistical significance, its magnitude is modest and unlikely to be clinically meaningful. Specifically, we have made the following changes:

** Abstract (Results) --- page 4, line 16: revised to "Weekend admissions showed a statistically significant but only modest reduction in mortality odds (OR: 0.99; 95% CI: 0.975-0.998)."

** Abstract (Conclusions) --- page 4, lines 22-24: revised to "Using a national cohort of hospitalized patients with decompensated cirrhosis, we showed that rural hospitals exhibited lower in-hospital mortality, fewer procedures, and higher transfer rates, and that weekend admissions showed only a minimal, clinically insignificant reduction in mortality, irrespective of hospital rurality."

** Results section --- page 17, line 5 (continuing): revised to "Weekend admissions were associated with a statistically significant but only modest reduction in the odds of in-hospital death compared to weekday admissions (OR 0.99, 95% CI: 0.975-0.998)."

** Discussion --- page 22, lines 25-30: the opening of the weekend-effect paragraph has been rewritten to explicitly frame the finding as the absence of a detrimental weekend effect rather than a true weekend survival advantage: "In this study, weekend admissions were associated with a statistically significant but only modest reduction in mortality (OR 0.99, 95% CI: 0.975-0.998). Given the magnitude of this effect (roughly a 1% relative reduction in odds across nearly 12 million hospitalizations), this finding is best interpreted as the absence of a detrimental weekend effect in contemporary cirrhosis care, rather than as a true weekend survival advantage, likely reflecting the effectiveness of standardized protocols for managing acute decompensation."

** Conclusions --- page 24, lines 23-25: revised to "Contrary to the traditional 'weekend effect,' weekend admissions were associated with a statistically significant but only modest reduction in mortality and shorter lengths of stay."

Attachment Submitted filename: R2_Response to the reviewers_PLOSONE_Rural_WE_R2.docx

# Peer review: Decision Letter 2

**DOI:** 10.1371/journal.pone.0353178.r005

**Authors:** Usama Waqar, Usama Waqar, Usama Waqar

10 May 2026

PONE-D-25-55717R2

Cirrhosis Outcomes on Rurality and Weekend Admissions Revisited: A Contemporary Analysis of the National Inpatient Sample

PLOS One

Dear Dr. Tanaka,

Thank you for submitting your manuscript to PLOS ONE. After careful consideration, we feel that it has merit but does not fully meet PLOS ONE’s publication criteria as it currently stands. Therefore, we invite you to submit a revised version of the manuscript that addresses the points raised during the review process.

Please find the editorial comments below.

Please submit your revised manuscript by Jun 24 2026 11:59PM. If you will need more time than this to complete your revisions, please reply to this message or contact the journal office at plosone@plos.org. When you're ready to submit your revision, log on to https://www.editorialmanager.com/pone/ and select the 'Submissions Needing Revision' folder to locate your manuscript file.

Please include the following items when submitting your revised manuscript:

- A letter that responds to each point raised by the academic editor and reviewer(s). You should upload this letter as a separate file labeled 'Response to Reviewers'.
- A marked-up copy of your manuscript that highlights changes made to the original version. You should upload this as a separate file labeled 'Revised Manuscript with Track Changes'.
- An unmarked version of your revised paper without tracked changes. You should upload this as a separate file labeled 'Manuscript'.

If you would like to make changes to your financial disclosure, please include your updated statement in your cover letter. Guidelines for resubmitting your figure files are available below the reviewer comments at the end of this letter.

If applicable, we recommend that you deposit your laboratory protocols in protocols.io to enhance the reproducibility of your results. Protocols.io assigns your protocol its own identifier (DOI) so that it can be cited independently in the future. For instructions see: https://journals.plos.org/plosone/s/submission-guidelines#loc-laboratory-protocols. Additionally, PLOS ONE offers an option for publishing peer-reviewed Lab Protocol articles, which describe protocols hosted on protocols.io. Read more information on sharing protocols at https://plos.org/protocols?utm_medium=editorial-email&utm_source=authorletters&utm_campaign=protocols.

As the corresponding author, your ORCID iD is verified in the submission system and will appear in the published article. PLOS supports the use of ORCID, and we encourage all coauthors to register for an ORCID iD and use it as well. Please encourage your coauthors to verify their ORCID iD within the submission system before final acceptance, as unverified ORCID iDs will not appear in the published article. *Only*  the individual author can complete the verification step; PLOS staff *cannot*  verify ORCID iDs on behalf of authors.

We look forward to receiving your revised manuscript.

Kind regards,

Usama Waqar, M.B.B.S

Academic Editor

PLOS One

Journal Requirements:

If the reviewer comments include a recommendation to cite specific previously published works, please review and evaluate these publications to determine whether they are relevant and should be cited. There is no requirement to cite these works unless the editor has indicated otherwise.

Please review your reference list to ensure that it is complete and correct. If you have cited papers that have been retracted, please include the rationale for doing so in the manuscript text, or remove these references and replace them with relevant current references. Any changes to the reference list should be mentioned in the rebuttal letter that accompanies your revised manuscript. If you need to cite a retracted article, indicate the article’s retracted status in the References list and also include a citation and full reference for the retraction notice.

Additional Editor Comments:

One of the key findings of the manuscript is that patients initially managed at rural hospitals had lower odds of mortality, while transfer rates from rural hospitals appear to be substantial. The authors should clarify how mortality is defined in this study, specifically whether it reflects in-hospital mortality only or includes a defined post-discharge follow-up period. The observed survival advantage in rural settings requires further clarification, as it is possible that a proportion of adverse outcomes occurred after transfer to urban centers. If mortality is not tracked across the full episode of care, including post-transfer outcomes, this could potentially bias comparisons between rural and urban hospitals.

[Note: HTML markup is below. Please do not edit.]

Reviewers' comments:

Reviewer's Responses to Questions

**Comments to the Author**

1. If the authors have adequately addressed your comments raised in a previous round of review and you feel that this manuscript is now acceptable for publication, you may indicate that here to bypass the “Comments to the Author” section, enter your conflict of interest statement in the “Confidential to Editor” section, and submit your "Accept" recommendation.

Reviewer #1: All comments have been addressed

**********

2. Is the manuscript technically sound, and do the data support the conclusions?

The manuscript must describe a technically sound piece of scientific research with data that supports the conclusions. Experiments must have been conducted rigorously, with appropriate controls, replication, and sample sizes. The conclusions must be drawn appropriately based on the data presented.

Reviewer #1: Yes

**********

3. Has the statistical analysis been performed appropriately and rigorously?

Reviewer #1: I Don't Know

**********

4. Have the authors made all data underlying the findings in their manuscript fully available?

The PLOS Data policy requires authors to make all data underlying the findings described in their manuscript fully available without restriction, with rare exception (please refer to the Data Availability Statement in the manuscript PDF file). The data should be provided as part of the manuscript or its supporting information, or deposited to a public repository. For example, in addition to summary statistics, the data points behind means, medians and variance measures should be available. If there are restrictions on publicly sharing data—e.g. participant privacy or use of data from a third party—those must be specified.

Reviewer #1: Yes

**********

5. Is the manuscript presented in an intelligible fashion and written in standard English?

PLOS ONE does not copyedit accepted manuscripts, so the language in submitted articles must be clear, correct, and unambiguous. Any typographical or grammatical errors should be corrected at revision, so please note any specific errors here.

Reviewer #1: Yes

**********

6. Review Comments to the Author

Please use the space provided to explain your answers to the questions above. You may also include additional comments for the author, including concerns about dual publication, research ethics, or publication ethics. (Please upload your review as an attachment if it exceeds 20,000 characters)

Reviewer #1: well done no further comments well done no further comments

well done no further comments thanks

**********

7. PLOS authors have the option to publish the peer review history of their article (what does this mean?). If published, this will include your full peer review and any attached files.

If you choose “no”, your identity will remain anonymous but your review may still be made public.

**Do you want your identity to be public for this peer review?**  For information about this choice, including consent withdrawal, please see our Privacy Policy.

Reviewer #1: No

**********

[NOTE: If reviewer comments were submitted as an attachment file, they will be attached to this email and accessible via the submission site. Please log into your account, locate the manuscript record, and check for the action link "View Attachments". If this link does not appear, there are no attachment files.]

To ensure your figures meet our technical requirements, please review our figure guidelines: https://journals.plos.org/plosone/s/figures

You may also use PLOS’s free figure tool, NAAS, to help you prepare publication quality figures: https://journals.plos.org/plosone/s/figures#loc-tools-for-figure-preparation.

NAAS will assess whether your figures meet our technical requirements by comparing each figure against our figure specifications.

# Author comment: Author response to Decision Letter 3

**DOI:** 10.1371/journal.pone.0353178.r006

12 May 2026

Response to the editorial suggestions:

Additional Editor Comments :

Comment: One of the key findings of the manuscript is that patients initially managed at rural hospitals had lower odds of mortality, while transfer rates from rural hospitals appear to be substantial. The authors should clarify how mortality is defined in this study, specifically whether it reflects in-hospital mortality only or includes a defined post-discharge follow-up period. The observed survival advantage in rural settings requires further clarification, as it is possible that a proportion of adverse outcomes occurred after transfer to urban centers. If mortality is not tracked across the full episode of care, including post-transfer outcomes, this could potentially bias comparisons between rural and urban hospitals.

RESPONSE: We thank the editor for this important observation. Our primary outcome is in-hospital mortality at the admitting facility only. The NIS does not link records across facilities, so post-transfer deaths are attributed to the receiving institution and cannot be traced back to the originating admission. We agree this could bias rural–urban comparisons. To address this concern, we have made the following revisions:

1. Methods (page 7, 3rd paragraph): Added an explicit definition clarifying that in-hospital mortality reflects death at the index facility and that post-transfer deaths are not linked back to the originating admission.

2. Discussion (page 22, 2nd paragraph): Added text acknowledging that transfer-related attribution could inflate urban mortality and deflate rural mortality, and noting that our sensitivity analyses excluding transfers showed an attenuated but persistent rural advantage (aOR 0.92 vs. 0.84).

3. Limitations (page 24, limitation section [1st paragraph]): Strengthened existing language to explicitly state that the NIS captures only index-facility mortality and that this design limitation may bias rural–urban comparisons when transfer patterns differ systematically

Attachment Submitted filename: R3_Response to the reviewers_PLOSONE_Rural_WE_R3.docx

# Peer review: Decision Letter 3

**DOI:** 10.1371/journal.pone.0353178.r007

**Authors:** Usama Waqar, Usama Waqar, Usama Waqar, Alessandro Granito

21 Jun 2026

Cirrhosis Outcomes on Rurality and Weekend Admissions Revisited: A Contemporary Analysis of the National Inpatient Sample

PONE-D-25-55717R3

Dear Dr. Tanaka,

We’re pleased to inform you that your manuscript has been judged scientifically suitable for publication and will be formally accepted for publication once it meets all outstanding technical requirements.

Within one week, you’ll receive an e-mail detailing the required amendments. When these have been addressed, you’ll receive a formal acceptance letter and your manuscript will be scheduled for publication.

An invoice will be generated when your article is formally accepted. Please note, if your institution has a publishing partnership with PLOS and your article meets the relevant criteria, all or part of your publication costs will be covered. Please make sure your user information is up-to-date by logging into Editorial Manager at Editorial Manager® and clicking the ‘Update My Information' link at the top of the page. For questions related to billing, please contact billing support.

If your institution or institutions have a press office, please notify them about your upcoming paper to help maximize its impact. If they’ll be preparing press materials, please inform our press team as soon as possible -- no later than 48 hours after receiving the formal acceptance. Your manuscript will remain under strict press embargo until 2 pm Eastern Time on the date of publication. For more information, please contact onepress@plos.org.

Kind regards,

Alessandro Granito

Academic Editor

PLOS One

Additional Editor Comments (optional):

Reviewers' comments:

Reviewer's Responses to Questions

**Comments to the Author**

1. If the authors have adequately addressed your comments raised in a previous round of review and you feel that this manuscript is now acceptable for publication, you may indicate that here to bypass the “Comments to the Author” section, enter your conflict of interest statement in the “Confidential to Editor” section, and submit your "Accept" recommendation.

Reviewer #1: All comments have been addressed

**********

2. Is the manuscript technically sound, and do the data support the conclusions?

The manuscript must describe a technically sound piece of scientific research with data that supports the conclusions. Experiments must have been conducted rigorously, with appropriate controls, replication, and sample sizes. The conclusions must be drawn appropriately based on the data presented.

Reviewer #1: Yes

**********

3. Has the statistical analysis been performed appropriately and rigorously?

Reviewer #1: I Don't Know

**********

4. Have the authors made all data underlying the findings in their manuscript fully available?

The PLOS Data policy requires authors to make all data underlying the findings described in their manuscript fully available without restriction, with rare exception (please refer to the Data Availability Statement in the manuscript PDF file). The data should be provided as part of the manuscript or its supporting information, or deposited to a public repository. For example, in addition to summary statistics, the data points behind means, medians and variance measures should be available. If there are restrictions on publicly sharing data—e.g. participant privacy or use of data from a third party—those must be specified.

Reviewer #1: Yes

**********

5. Is the manuscript presented in an intelligible fashion and written in standard English?

PLOS ONE does not copyedit accepted manuscripts, so the language in submitted articles must be clear, correct, and unambiguous. Any typographical or grammatical errors should be corrected at revision, so please note any specific errors here.

Reviewer #1: Yes

**********

6. Review Comments to the Author

Please use the space provided to explain your answers to the questions above. You may also include additional comments for the author, including concerns about dual publication, research ethics, or publication ethics. (Please upload your review as an attachment if it exceeds 20,000 characters)

Reviewer #1: No further comments . The authors have responded to all comments and improved their manuscript. Good luck

**********

7. PLOS authors have the option to publish the peer review history of their article (what does this mean?). If published, this will include your full peer review and any attached files.

If you choose “no”, your identity will remain anonymous but your review may still be made public.

**Do you want your identity to be public for this peer review?**  For information about this choice, including consent withdrawal, please see our Privacy Policy.

Reviewer #1: No

**********

# Editor report: Acceptance letter

**DOI:** 10.1371/journal.pone.0353178.r008

**Authors:** Usama Waqar, Usama Waqar, Usama Waqar, Alessandro Granito

PONE-D-25-55717R3

PLOS One

Dear Dr. Tanaka,

I'm pleased to inform you that your manuscript has been deemed suitable for publication in PLOS One. Congratulations! Your manuscript is now being handed over to our production team.

At this stage, our production department will prepare your paper for publication. This includes ensuring the following:

* All references, tables, and figures are properly cited

* All relevant supporting information is included in the manuscript submission,

* There are no issues that prevent the paper from being properly typeset

You will receive further instructions from the production team, including instructions on how to review your proof when it is ready. Please keep in mind that we are working through a large volume of accepted articles, so please give us a few days to review your paper and let you know the next and final steps.

Lastly, if your institution or institutions have a press office, please let them know about your upcoming paper now to help maximize its impact. If they'll be preparing press materials, please inform our press team within the next 48 hours. Your manuscript will remain under strict press embargo until 2 pm Eastern Time on the date of publication. For more information, please contact onepress@plos.org.

You will receive an invoice from PLOS for your publication fee after your manuscript has reached the completed accept phase. If you receive an email requesting payment before acceptance or for any other service, this may be a phishing scheme. Learn how to identify phishing emails and protect your accounts at https://explore.plos.org/phishing.

If we can help with anything else, please email us at customercare@plos.org.

Thank you for submitting your work to PLOS ONE and supporting open access.

Kind regards,

PLOS ONE Editorial Office Staff

on behalf of

Dr. Alessandro Granito

Academic Editor

PLOS One
